# Home-Based, Virtually Supervised Combined Exercise Intervention in People With Parkinson Disease: Protocol for a Randomized Controlled Trial

**DOI:** 10.2196/97507

**Published:** 2026-06-30

**Authors:** Niyati Mehta, Miranda J Munoz, Isabel E Allen, Caroline M Tanner, Daniel M Corcos, Nijee S Luthra

**Affiliations:** 1 Department of Physical Therapy and Human Movement Sciences, Northwestern University, Chicago, IL, United States; 2 Department of Epidemiology, Data Science, and Biostatistics, University of California, San Francisco, San Francisco, CA, United States; 3 Global Brain Health Institute, University of California, San Francisco, San Francisco, CA, United States; 4 Department of Neurology, Weill Institute for Neurosciences, University of California, San Francisco, 1651 4th Street, San Francisco, CA, 94158, United States, 1 415-353-2311; 5 Parkinson's Elevated, Intermountain Health, Salt Lake City, UT, United States

**Keywords:** Parkinson disease, endurance exercise, resistance exercise, treadmill exercise, weight training, cognition, blood biomarkers

## Abstract

**Background:**

Cognitive impairment begins early in Parkinson disease (PD) and progresses to dementia in most people with PD, reducing quality of life and contributing to growing health-related costs. Physical exercise has potent antiaging effects and improves many outcomes in PD, including cognition. Identifying biomarkers that respond to exercise and determining how they associate with cognition and underlying disease pathology may elucidate key mechanisms for countering cognitive decline.

**Objective:**

This clinical trial will test the feasibility, adherence, and safety of a 26-week home-based, combined endurance and resistance exercise intervention in people with PD. Secondary objectives are to test the effects of the exercise intervention on (1) global cognition, (2) motor symptom progression, and (3) circulating fluid-based biomarker levels.

**Methods:**

The Exercise for Cognitive Excellence in Parkinson’s Disease study primarily evaluates the feasibility, adherence, and safety of a home-based exercise intervention in people with PD. It is secondarily a pilot randomized controlled trial that measures the effect of this intervention on cognition, motor progression, and circulating biomarkers. Thirty-one participants with PD will be randomized to either a home-based, trainer-supervised endurance and resistance training program (exercise group) or a waitlist control group for 26 weeks. Feasibility will be assessed using the average percentage of maximum heart rate (HR) for aerobic exercise and repetition maximums for resistance exercise. Adherence will be assessed using average days of exercise per week, average duration of exercise at target HR intensity for aerobic exercise, and average duration of exercise for resistance exercise. Safety will be assessed by measuring the number of adverse events and serious adverse events. The efficacy of the combined endurance and resistance exercise intervention will be measured using cognitive assessments, the Movement Disorders Society Unified Parkinson’s Disease Rating Scale, and participant-reported outcomes, all obtained at baseline and 26 weeks. Biomarkers in the periphery (blood and saliva) and brain (cerebrospinal fluid) will also be measured before and after the 26-week exercise intervention.

**Results:**

Recruitment commenced in July 2023 and concluded in November 2025. The last participant will complete data collection in May 2026. Data will be analyzed starting in June 2026, and results are expected to be published in late 2026 and early 2027.

**Conclusions:**

Previous studies have shown that high-intensity endurance exercise effectively slows the progression of motor symptoms in PD and that resistance exercise effectively improves cognition in PD. Establishing whether a clinically relevant, combined endurance and resistance exercise intervention is safe and feasible in PD and can improve cognition and slow motor disease progression would have a significant impact on quality of life for those with PD and their caregivers. Understanding how biomarkers respond to exercise will shed important mechanistic insight.

## Introduction

### Background and Rationale

With the world’s population aging, the number of people with Parkinson disease (PD) is expected to rise to 14 million by 2040 [1,2]. While PD is primarily characterized as a movement disorder, it is a multidomain disease, and a substantial proportion of people with PD are burdened with nonmotor symptoms, including cognitive dysfunctions. Mild cognitive impairment is now recognized in 15%‐20% of people with newly diagnosed, early PD [3,4] and is evident in up to 60% of individuals after diagnosis [5]. As the disease progresses, the cumulative incidence of dementia rises to 75%‐90% [5-7]. Cognitive decline is associated with poorer quality of life, greater caregiver burden, increased risk of nursing home placement, and growing health-related costs [8-10].

There are numerous treatments for motor symptoms in PD, including anti-Parkinson medications, deep brain stimulation, and various modes of exercise, but there are no effective pharmacological or surgical treatments for cognitive impairments in PD [11]. While multiple medications have been studied for potential benefits to cognition in PD, only one has shown any benefit for PD dementia alone, and it is associated with side effects such as nausea, vomiting, and tremors [11]. For the larger population of people with PD with nondementia cognitive impairments, the effects were inconclusive [11]. Evidence has also shown that deep brain stimulation can even worsen cognition [12-14]. Therefore, there is a great need for interventions that benefit cognition for people with PD. Encouragingly, exercise has been widely shown to be effective at improving cognition in healthy aging individuals [15,16]. While there is less evidence in people with PD, studies have typically found a benefit of exercise on cognition in people with PD, in addition to the well-established benefits of exercise on motor signs and disease progression [17-21]. Additionally, exercise is simple and safe to implement, and it has widespread benefits on multiple body systems [22].

To examine the effects of exercise on the body and brain, we will use fluid biomarker measurements from both the central nervous system and the periphery. Analyzing biomarkers that respond to exercise could offer valuable insights into the mechanisms by which exercise impacts cognition in PD. In healthy individuals, exercise has been shown to increase neurotrophic factors (ie, brain-derived neurotrophic factor [BDNF]), decrease inflammatory markers (ie, C-reactive protein [CRP]), and influence neuroendocrine markers of aging (ie, increased klotho) and stress (ie, decreased cortisol) [23]. People with PD may even have a magnified response to exercise because these markers are impacted by the disease and tied to cognitive impairments. Although not all these markers have been examined in PD specifically, current evidence shows a relationship between these biomarkers and cognition. In PD, studies have shown that there is reduced BDNF, increased CRP, reduced klotho, and elevated cortisol [23]. In PD specifically, BDNF levels have been shown to negatively correlate with both age of PD onset and cognitive dysfunction [24], and elevated CRP in the cerebrospinal fluid (CSF) is associated with cognitive impairments in PD [25]. Although less studied in PD, studies have shown that higher cortisol is associated with cognitive dysfunction [26], while higher klotho levels are associated with improved cognition in healthy adults [27]. While it has been shown that exercise influences these markers, they are affected in PD, and that they are related to cognition, the effects of exercise on these markers on cognition in PD are still unknown.

Although there is a growing consensus that exercise is beneficial for both cognitive and motor symptoms of PD [17,19-21], most studies thus far have focused on a single exercise modality. For this study, we chose a novel combined intervention of high-intensity endurance exercise and resistance training as it provides more clinical relevance by targeting both the cardiorespiratory and musculoskeletal systems [28]. We also chose a virtually supervised intervention as it allows participants to complete the exercise intervention from their own home, which increases access and long-term adherence to exercise, as it limits the need for people with PD to leave their homes [29]. The use of the combined endurance and resistance exercise intervention, delivered at home, will allow us to better understand the clinical implications of exercise on cognition, motor symptoms, and fluid-based biomarkers in a context applicable to everyday patients with PD outside of the lab.

This trial will primarily test the feasibility, adherence, and safety of a home-based, virtually supervised, combined high-intensity endurance and resistance training program. It is also a pilot randomized controlled trial to evaluate the efficacy of the combined intervention on cognition, motor signs, and underlying biomarkers in the periphery and brain. The outcome of this trial will (1) demonstrate the feasibility of a new combined training program and (2) provide preliminary evidence for how this clinically relevant exercise intervention can improve cognition and motor signs in PD and how it associates with biomarker changes.

### Explanation for the Choice of Comparator

The exercise intervention for this study is a combined endurance and resistance exercise intervention. This intervention was chosen as it has high clinical relevance–an ideal exercise prescription for PD includes both exercises to target the cardiopulmonary and musculoskeletal systems [28]. For this combined intervention, the frequency of endurance exercise is 3 times per week, and resistance exercise is 2 times per week. The endurance exercise protocol was modeled after the Study in Parkinson’s Disease of Exercise Phase III clinical trial [30]. Endurance exercise will be performed at a high intensity with a target HR zone of 80%‐85% of maximal heart rate (HRmax). Treadmill exercise was chosen as it allows participants to follow a regular rhythmic stimulus and allows participants to keep a consistent speed and grade, which should help maintain HR in the target zone. The 80%‐85% HRmax zone is calculated using a measured HRmax from a baseline cardiopulmonary exercise test. Using a measured HRmax instead of estimating with equations is particularly important in PD, as there may be increased prevalence of autonomic dysfunction affecting HRmax in PD [31]. The resistance training protocol chosen for this study is modeled after a resistance training program in PD participants with similar disease stage and study duration as our proposed study [32].

The control group was chosen to test the hypothesis that the combined endurance and resistance exercise intervention benefits cognition and motor signs of PD more than best medical management, which usually involves prescribing anti-Parkinson medication but no exercise. Few studies have looked at a combined endurance and resistance exercise intervention in PD. This study will demonstrate how a combined intervention may benefit cognitive and motor signs of the disease and influence circulating biomarkers compared to usual care.

### Objectives

This clinical trial will primarily test the feasibility, adherence, and safety of a 26-week combined endurance and resistance exercise intervention in people with PD. Secondary objectives are to test the effects of the exercise intervention on (1) global cognition, as measured by the Montreal Cognitive Assessment (MoCA), (2) the progression of motor symptoms as measured by the Movement Disorders Society Unified Parkinson’s Disease Rating Scale (MDS-UPDRS) motor score (part III), and (3) circulating neurotrophic (BDNF), inflammatory (CRP), and neuroendocrine (klotho and cortisol) biomarkers in blood, saliva, and CSF. Exploratory objectives are to test the effects of exercise on (1) domain-specific cognitive functions including executive function, attention and working memory, visuospatial function, memory, and language; (2) various measures of fitness and motor function; and (3) participant-reported outcomes of quality of life, mood, and fatigue.

## Methods

### Ethical Considerations

#### Ethics Approval and Consent to Participate

The Exercise for Cognitive Excellence in Parkinson’s Disease (EXCEL-PD) trial has been approved by the University of California, San Francisco (UCSF) Institutional Review Board (IRB; protocol number 22-38258) in accordance with the Declaration of Helsinki. Written informed consent to participate in the study is obtained from all participants.

#### Informed Consent

Standard written informed consent is obtained at screening prior to starting the study procedures. The institution’s HIPAA (Health Insurance Portability and Accountability Act) form will also be reviewed and signed by each participant at the time of the informed consent. The consent will be explained to the participant in detail by the study investigator, including but not limited to the study purpose, duration, procedures, risks, benefits, confidentiality, instructions on whom to contact with questions, and statements that participation is voluntary. The study team will ensure that information is given in a language understandable to the participant and their questions will be answered. We will not permit consent from a legally authorized representative for this study. We will not assess capacity to consent explicitly. Referring neurologists will only refer people whom they think can consent. Comprehension and autonomy will be assessed by asking participants to explain in their own words what the study is about.

#### Confidentiality

Personal identifying information on forms will only be included when necessary (ie, consent forms), and all data will be deidentified. Study data will be stored in a secure location at UCSF, and interviews and collection of personal information will be conducted in a private setting.

All biological samples will be deidentified and labeled with a unique participant identifier to prevent tracing back to the participant.

If the participant is referred for urgent psychiatric evaluation for suicidal thinking identified during routine study monitoring and mood questionnaire (Beck Depression Inventory-II [BDI-II]), they will be referred to their PCP for further evaluation and management of depression. If suicidal thinking was identified at screening, they will be excluded from the study. If suicidal screening is identified at a subsequent study visit, it will be treated as an AE and followed to record the date of resolution and outcome.

### Trial Design

**Figure 1.**
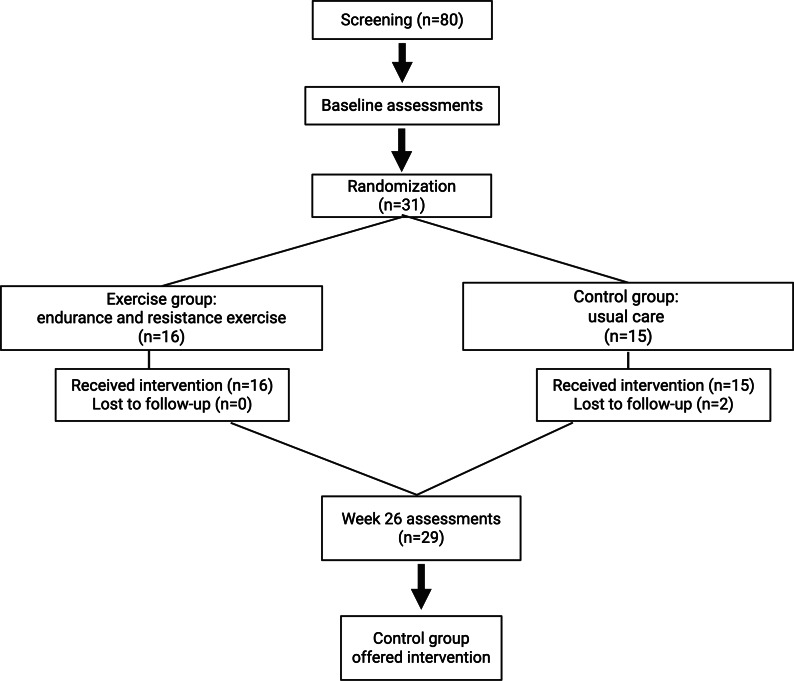
Design of the Exercise for Cognitive Excellence in Parkinson’s Disease Trial.

EXCEL-PD is a single-site, randomized controlled clinical trial to study the feasibility, adherence, and safety of a home-based, virtually supervised exercise program and test the hypothesis that a combined high-intensity endurance and resistance exercise intervention improves cognition and slows the progression of motor symptoms compared to a control group. Assessments occur at baseline and 26 weeks (Figure 1).

### Trial Setting

This is a single-center study being conducted at the UCSF in San Francisco, California. UCSF has an affiliated movement disorders clinic, which will be the primary site of recruitment. All study procedures are conducted at UCSF, and all data and specimens will be stored at UCSF.

### Eligibility Criteria for Participants

Our study population is defined as persons 40‐80 years of age with a diagnosis of idiopathic PD and a modified Hoehn and Yahr stage less than 4. If they are treated with PD medications, they must have stable doses for at least 2 months prior to baseline. If they are untreated with PD medications at baseline, they must be deemed unlikely to require medication for the next 6 months. Potential participants undergo a telephone prescreening. If eligible, they come to the study site for informed consent and a screening visit (Textbox 1).

Textbox 1. **Inclusion and exclusion criteria for Exercise for Cognitive Excellence in Parkinson Disease trial.**

**Inclusion criteria**

- Diagnosis of idiopathic Parkinson disease (PD) based on Movement Disorder Society criteria, with bradykinesia plus one of the other cardinal signs of PD (resting tremor and rigidity), without any other known or suspected cause of parkinsonism.
- Modified Hoehn and Yahr stage less than 4
- Age 40-80 years at the time of screening
- Able to provide informed consent
- Willing to comply with the study protocol
- If being treated with PD symptomatic medications (ie, rasagiline, carbidopa and levodopa, dopamine agonists, amantadine, and anticholinergics), stable doses for 2 months prior to baseline. If not being treated with PD symptomatic medication at the time of screening, deemed unlikely to require symptomatic medication for the next 6 months.

**Exclusion criteria**

- A diagnosis of atypical parkinsonism, drug-induced parkinsonism, essential tremor, primary dystonia, or other diagnoses that explain symptoms other than PD.
- A diagnosis of a significant neurological disease other than PD that would interfere with the ability to perform study procedures or assessments.
- Significant cognitive impairment is defined as Montreal Cognitive Assessment <23 or any impairment that would, in the opinion of the investigator, interfere with the ability to follow exercise directions.
- History of frequent falls (ie, falling multiple times per week) or considered high fall risk based on investigator assessment.
- Beck Depression Inventory-II score >28, indicating severe depression that precludes the ability to exercise. Any participant with such a score will be referred to a primary care physician (PCP) or physician for further evaluation and management of depression. Individuals with a Beck Depression Inventory-II score of 17‐28 will be excluded if any of the following conditions are met: the individual is suicidal, the individual is currently in need of depression treatment modification, or depressive symptoms are likely to interfere with adherence to the study protocol. Any participant with such a score will be referred to a PCP or physician for further evaluation and management of depression.
- Recent use of psychotropic medications (ie, anxiolytics, hypnotics, benzodiazepines, and antidepressants) where dosage has not been stable for 30 days prior to screening.
- Presence of known cardiovascular, metabolic, or renal disease or individuals with major signs or symptoms suggestive of cardiovascular, metabolic, or renal disease without medical clearance to participate in the exercise program.
- Presence of any of the following laboratory abnormalities on screening labs:
  - Abnormal liver function (aspartate aminotransferase or alanine aminotransferase more than 2 times the upper limit of normal).
  - Abnormal renal function (creatinine clearance calculated by the Cockcroft-Gault equation <50 mL/min or estimated glomerular filtration rate using the MDRD4 equation or the CKD-EPI [Chronic Kidney Disease Epidemiology Collaboration] equation <45 mL/min/1.73 m^2^).
  - Complete blood count out of range on screening labs and physician’s judgment that the abnormal value is clinically significant.
- Uncontrolled hypertension (resting blood pressure >150/90 mm Hg).
- Orthostatic hypotension and standing systolic blood pressure below 100. Orthostatic hypotension is a reduction of systolic blood pressure of at least 20 mm Hg or diastolic blood pressure of at least 10 mm Hg within 3 minutes of standing.
- Already participating in 120 minutes or more of moderate-intensity exercise per week.
- Serious illness (requiring systemic treatment and/or hospitalization) within the last 4 weeks.
- History of any other medical problem or injury that may interfere with the ability to exercise.
- Conditions that preclude the safe performance of routine lumbar puncture, including
  - international normalized ratio >1.4 or other coagulopathy
  - platelet cell count of <50,000/μL
  - infection at the desired lumbar puncture site
  - taking anticoagulant medication within 90 days of baseline (note: low-dose aspirin is permitted)
  - suspected noncommunicating hydrocephalus or intracranial mass
  - prohibitive lumbar spinal disease
- Enrollment in another investigational study that includes an intervention; participation in noninterventional studies may be permitted.
- Receipt of any non-PD investigational product or device or participation in a non-PD drug research study within a period of 30 days (or 5 half-lives of the drug, whichever is longer) before baseline.
- Lack of access to a computer or tablet and Wi-Fi or any other technical challenges that, in the opinion of the investigator, would prevent participation in the virtually supervised exercise training program.

### Intervention and Comparator

Participants in the exercise group will receive 26 weeks of a home-based, virtually supervised combined endurance and resistance training program. Participants will be provided with required exercise equipment to allow them to exercise at home, including a treadmill, free weights, an exercise bench, and a mat. Exercise equipment will be delivered directly to a participant’s residence for in-home use.

Virtually supervised sessions will be delivered using the Zoom platform (Zoom Video Communications, Inc) by the Movement Revolution, Inc team of Neuro Exercise Specialists (Chicago, IL). All sessions during weeks 1‐2 will be supervised, and they will consist of familiarization sessions led by a Neuro Exercise Specialist who will educate participants on correct and safe use of exercise equipment, including a treadmill and weights. The Neuro Exercise Specialist will review how to get on and off the treadmill safely, how to use handrails on treadmills to assist with balance if needed, and correct form for lifting weights. Participants will also be instructed not to multitask or have distractions around that could increase the risk of falls and never to exercise when feeling lightheaded or dizzy or when Parkinson symptoms are worse than usual. By the end of week 2, trainers will ensure that participants can exercise safely and independently before reducing the number of supervised sessions. If participants or the Neuro Exercise Specialist still have concerns for safety after the initial 2 weeks of training sessions, participants will continue supervised virtual sessions or come to the research center for in-person exercise supervision.

Weeks 3‐6 will include 10 supervised sessions and a ramp-up phase that will include gradual increases in HR for endurance exercise and weights and repetitions for resistance exercises. If a participant is unable to do a particular exercise, the trainer will make modifications as appropriate. By the end of week 6, participants will be exercising 5 days per week (3 days of endurance training and 2 days of resistance training) at their prescribed endurance and resistance intensities. Weeks 7‐26 will be the full intervention and will include 1 supervised session per week.

Endurance training will be performed 3 times per week on a treadmill to target 80%‐85% of their HRmax for a duration of 40 minutes (30 minutes on the treadmill at target HR with 5 minutes each for warm-up and cool-down).

Participants will be provided with an HR monitor (Polar H10), paired with the Polar Beat app (Polar) on their smartphone, to allow tracking of HR and activity by the participant and investigative team. The study team will review and download HR data for each exercise session and enter it into a secure electronic database. Participants will ramp up their exercise intensity to achieve their target HR zone over the first 6 weeks of the study. Endurance exercise will begin at 60% HRmax and progressively increase to 80%‐85% HRmax by week 6. Participants will be instructed on monitoring HR and adjusting the exercise intensity to remain in the target HR zone by changing treadmill speed and/or incline. Weekly phone calls will coach participants on adjustments to continue exercising at 80%‐85% HRmax.

Resistance training will be performed 2 times a week, with sessions separated by 48 hours. Resistance exercise sessions should be about 1 hour in length but will vary by participant. One repetition maximum (RM) will be calculated for each participant using the following formula: 1RM=weight × (1+0.0333 × repetitions). Participants will start with a training load that is 60% of 1RM. The full volume exercise prescription will consist of 5 minutes of dynamic warm-up followed by 5 exercises using free weights to improve strength and muscle mass (performed for 3 sets of 12‐20 repetitions) and one isometric strength exercise for core stabilization (performed for 1 minute). Exercises will be divided into 2 circuits: one will focus on arms, back, and core, while the other will focus on legs and core. After the first 13 weeks, resistance exercises will be changed in each circuit to continue to challenge participants, but they will still target the same muscle groups (Table S1 in Multimedia Appendix 1).

A sample training schedule is included (Table S2 in Multimedia Appendix 2), but this will be tailored based on participants’ progress. Supervision will take place until the participant is deemed independent. Participants will be asked to demonstrate all steps of the exercise regimen on the treadmill in the last supervised sessions before being deemed independent, and they will be asked to repeat this at each subsequent supervised session, taking place once a week over the duration of the study. All participants will be offered as much support as needed to successfully complete the exercise intervention. The study team will offer additional phone calls, supervised sessions, or in-person assistance at the research center. Video tutorials will be made available to all participants as guides for using the exercise equipment and for being able to visualize the resistance training exercise movement patterns.

Participants in the usual care control group will continue their baseline level of activity that they were doing prior to enrollment in the study and will be asked to complete physical activity logs to evaluate adherence. At the end of the randomization period, participants in the control group will be offered the chance to participate in the 26-week home-based, combined endurance and resistance training program.

### Criteria for Discontinuing or Modifying Allocated Intervention and Comparator

If a participant decides to discontinue the exercise intervention, they will be asked to return for a follow-up visit to complete the week 26 assessments. The reason for discontinuation will be documented. Participants are free to withdraw from participation in the study at any time upon request. An investigator may discontinue a participant from the study, including but not limited to the following reasons: lost to follow-up, unable to contact participant, any event, medical condition, or situation occurs such that continued collection of follow-up study data would not be in the best interest of the participant, or the participant meets an exclusion criterion (either newly developed or not previously recognized) that precludes exercise. In this scenario, the participant would still be followed for outcomes.

If a participant is unable to complete a specific exercise due to injury or discomfort, but still wants to complete the intervention, the Neuro Exercise Specialists will adapt the exercises for that participant while still targeting the intended muscle group as best as possible.

### Strategies to Improve Adherence to Intervention and Comparator

Several measures are taken to improve and monitor adherence. First, participants will be wearing an HR monitor during every endurance exercise session, which will provide them with visual feedback regarding their current HR and exercise duration. The HR monitor will record both the HR and the duration of each exercise session. Participants will be asked to upload the HR monitor data weekly to a secure link so the study team can review and archive it. Second, participants will also be given an exercise diary to record each exercise session. Third, participants will have weekly supervised sessions that will provide accountability and oversight to help with adherence and ensure safe and appropriate use of exercise equipment. Finally, the investigative team will call the participant weekly to review their average HR during exercise, the duration of exercise, and the number of days exercised. If a participant is not adhering to the targets for HR, duration, and frequency, the investigative team and exercise trainers will discuss strategies with the participant to reach appropriate exercise targets.

### Concomitant Care Permitted or Prohibited During the Trial

During this study, any new medications or changes to medication dosage during the study period will be reviewed weekly over the phone. Additionally, participants in the waitlist control group are asked to refrain from increasing their activity level for the duration of the study and keep a physical activity log to track any activities they participate in during the study. Participants are also asked to refrain from enrolling in any other interventional studies.

### Ancillary and Posttrial Care

Given the nature of the intervention, there are no provisions for posttrial care. Participants may keep all home exercise equipment given to them for personal use. Participants in the waitlist control group will be offered the intervention at the conclusion of the study.

### Outcomes

#### Overview

The outcomes for EXCEL-PD are listed below as well as in Table 1, which includes the domain, measure, metric, method of aggregation, and time point for each outcome.

**Table 1.** EXCEL-PD^a^ trial outcomes.

Outcome measure	Domain	Specific measurement variable	Metric	Method of aggregation	Time points
Primary outcomes					
Feasibility	Feasibility	Average % HRmax^b^ and RM^c^	Value at time point assessed	Mean, SD, median, range	Weeks 7‐26
Adherence	Adherence	Number of days exercised per week and duration at target intensity per session	Value at time point assessed	Mean, SD, median, range	Weeks 7‐26
Safety	Safety	Incidence of adverse events and serious adverse events	Number of events	Number and rate of events	Continuous
Secondary outcomes					
Global cognitive function	Cognitive function	Montreal Cognitive Assessment Scale	Score at time point assessed	Mean, SD, median, range	Screening and 26 weeks
MDS-UPDRS^d^ motor score (part III)	Motor sign assessment of Parkinson disease	MDS-UPDRS motor score (part III)	Score at time point assessed	Mean, SD, median, range	Baseline and 26 weeks
Klotho	Neuroendocrine	Klotho protein (nm/L)	Value at time point assessed	Mean, SD, median, range	Baseline and 26 weeks
Cortisol	Neuroendocrine	Cortisol hormone (pg/mL)	Value at time point assessed	Mean, SD, median, range	Baseline and 26 weeks
Brain-derived neurotrophic factor	Neurotrophic	BDNF^e^ protein (pg/mL)	Value at time point assessed	Mean, SD, median, range	Baseline and 26 weeks
C-reactive protein	Inflammation	CRP^f^ protein (mg/L)	Value at time point assessed	Mean, SD, median, range	Baseline and 26 weeks
Exploratory outcomes					
Domain-specific cognitive function	Cognitive function	Trail Making Test, Alternating Verbal Fluency, and NIH^g^ Toolbox	Score at time point assessed	Mean, SD, median, range	Baseline and 26 weeks
Six-minute walk	Functional capacity	Distance walked in 6 minutes in meters	Value at time point assessed	Mean, SD, median, range	Baseline and 26 weeks
VO2peak^h^	Cardiorespiratory fitness	Peak volume of oxygen consumed	Value at time point assessed	Mean, SD, median, range	Baseline and 26 weeks
Hand grip and knee extensor strength	Strength	Maximum strength (kg)	Value at time point assessed	Mean, SD, median, range	Baseline and 26 weeks
Participant-reported outcomes	Quality of life, depression, anxiety, apathy, fatigue, loneliness	The PDQ-39^i^, BDI-II^j^, PAS^k^, AS^l^, PFS-16^m^, UCLA^n^ Loneliness Scale	Score at time point assessed	Mean	Baseline and 26 weeks
Experiences of daily living	Experiences of daily living	MDS-UPDRS part I and II, PDAQ-15^o^	Score at time point assessed	Mean, SD, median, range	Baseline and 26 weeks
Global Impression of Change	Clinical outcome assessment	PGI-C^p^, CGI-C^q^	Score at time point assessed	Mean, SD, median, range	26 weeks

a EXCEL-PD: Exercise for Cognitive Excellence in Parkinson’s Disease.

b HRmax: maximal heart rate.

c RM: repetition maximum.

d MDS-UPDRS: Movement Disorders Society Unified Parkinson’s Disease Rating Scale.

e BDNF: brain-derived neurotrophic factor.

f CRP: C-reactive protein.

g NIH: National Institutes of Health.

h VO2peak: maximum volume of oxygen consumption.

i PDQ-39: Parkinson’s Disease Questionnaire-39.

j BDI-II: Beck Depression Inventory-II.

k PAS: Parkinson Anxiety Scale.

l AS: Apathy Scale.

m PFS-16: Parkinson’s Disease Fatigue Scale.

n UCLA: University of California, Los Angeles.

o PDAQ-15: Parkinson’s Daily Activities Questionnaire-15.

p PGI-C: Patient Global Impression of Change.

q CGI-C: Clinical Global Impression of Change.

#### Primary Outcomes

The primary outcomes for the study are feasibility, adherence, and safety of the combined exercise intervention. Adherence and feasibility will only be evaluated in the exercise group. Feasibility will be measured using the average percentage of HRmax for endurance exercise sessions and the average repetition maximum for resistance exercise sessions. Adherence will be measured using the average number of days exercised per week, the duration of exercise for resistance exercise, and the duration of exercise at target HR intensity for endurance exercise. Participants will keep track of exercise sessions using exercise diaries, which investigators will use to assess adherence. Data from Polar HR monitors will be used to assess feasibility. Safety will be measured by the incidence of adverse events (AEs) and serious AEs in the exercise and control groups.

#### Secondary Outcomes

The secondary outcomes will be the effects of the combined exercise intervention on global cognition, motor symptoms severity, and biomarker levels. These outcomes will be measured at baseline and at week 26 postintervention for both the exercise and control groups. Global cognition will be assessed using MoCA. Motor symptoms severity will be assessed using the MDS-UPDRS motor score (part III). Changes in neurotrophic (BDNF), inflammatory (CRP), and neuroendocrine (klotho and cortisol) biomarker levels will be assessed using blood, saliva, and CSF samples.

#### Exploratory Outcomes

The study will collect various other measures that may respond to exercise. This includes a cognitive battery, various measures of motor function and fitness, and participant-reported outcomes.

1. Cognitive battery: paper-based assessments (Trail Making Test and Alternating Verbal Fluency) and tablet-based assessments (NIH [National Institutes of Health] Toolbox) will be performed. Cognitive categorization will also be completed by the investigator to assess if the participant has normal cognition, mild cognitive impairment, or dementia.
2. 6-minute walk test: participants will be asked to walk for 6 minutes at a comfortable speed. The distance walked will be measured.
3. Maximum volume of oxygen consumption (VO2peak): under the direction of a trained exercise physiologist, participants will walk on a treadmill beginning at 0% grade at a speed that elicits approximately 70% of their age-predicted HRmax. The incline of the treadmill will increase by 2% every 2 minutes until either the participant reaches severe fatigue that forces cessation of exercise or if any of the end points of exercise testing are met. The end points that will be used to stop the exercise test before volitional fatigue include the development of (1) chest pain or discomfort; (2) a fall of systolic blood pressure of 10 mm Hg or greater from the peak level with increasing exercise intensity; (3) excessive rise in blood pressure: diastolic blood pressure >115 mm Hg and/or systolic >220 mm Hg; (4) signs of poor perfusion: light-headedness, confusion, ataxia, pallor, cyanosis, nausea, or cold and clammy skin; (5) shortness of breath, wheezing, leg cramps, or claudication; (6) failure of HR to increase with increased exercise intensity; (7) participant requests to stop; and (8) failure of testing equipment. The VO2peak will be measured and used as an indicator of fitness level.
4. Hand-grip and knee extensor strength testing: handgrip strength will be measured using a handheld digital dynamometer, and knee extensor strength will be measured using isokinetic dynamometry.
5. Participant-reported outcomes of quality of life, fatigue, and mood: the Parkinson’s Disease Questionnaire-39 (PDQ-39), Parkinson’s Disease Fatigue Scale (PFS-16), Parkinson Anxiety Scale (PAS), Apathy Scale (AS), University of California, Los Angeles (UCLA) Loneliness Scale, and BDI-II will be completed. The PDQ‐39 is a self-report questionnaire of 39 items, anchored on a 5‐point Likert scale, to assess PD-specific health‐related quality of life. The PFS-16 was designed to tap a single construct encompassing the physical aspects of fatigue and their impact on the participant’s daily function. The PAS is a 12-item observer or participant-rated scale with 3 subscales for persistent anxiety, episodic anxiety, and avoidance behavior that provides a reliable measurement of the severity of anxiety symptoms in patients with PD. The AS consists of 14 items regarding different dimensions of apathetic behavior. The UCLA Loneliness Scale consists of 20 items designed to measure feelings of loneliness and social isolation. The BDI-II consists of 21 items that are designed to assess the severity of symptoms of depression.
6. Experiences of daily living: the MDS-UPDRS Part I and II are used to evaluate various aspects of PD, including nonmotor and motor experiences of daily living and motor complications. Penn’s Parkinson’s Daily Activities Questionnaire-15 (PDAQ-15): the PDAQ-15 is an item-response theory-based questionnaire that assesses cognitive instrumental activities of daily living.
7. Patient Global Impression of Change: this is a 7-point self-report questionnaire depicting the patient’s rating of overall improvement.
8. Clinician’s Global Impression of Change: this is a 7-point clinician-rated measure depicting the clinician’s rating of overall improvement.

### Harms

Overall, the potential risks associated with participation in this study are of low risk. We do not anticipate any harm from this study; however, possible harms include muscle soreness or strains, falls, and pain or bruising associated with blood draws and lumbar puncture. The study investigator will be responsible for determining whether an AE is expected or unexpected. An AE will be considered unexpected if the nature, severity, or frequency of the event is not consistent with the risk information previously described for the study procedures and provided in the consent form. All harms will be documented, and any AEs occurring on site that are serious or unexpected AND definitely, probably, or possibly related to the study will be reported to the UCSF IRB within 5 working days. All AEs will be reported in the trial publication.

### Participant Timeline

The complete schedule of activities for participants is provided in Multimedia Appendix 3. This includes prescreening, screening and baseline visits, weekly phone calls, and week 26 visits. Assessments for each visit and intervention period are also noted. Additional visits will be scheduled as needed, such as if there are safety concerns with the exercise intervention.

### Sample Size

We based the sample size calculations on the secondary outcomes of efficacy. Since adherence and feasibility can only be evaluated in the exercise group and are not collected in the control group, it is not possible to calculate power comparing these outcomes between groups. The power and sample size analysis is based on the biomarker of interest, klotho, and the global cognition measure, MoCA. Based on prior studies describing changes in klotho plasma levels with exercise in healthy sedentary adults [33,34], a sample size of 14 participants per group is expected to detect an effect size of 100  pg/mL, considering a type I error of 0.05 with a statistical power of 0.85. Assuming a maximum attrition rate of 12%, we decided to recruit a total of 31 participants (16 participants in the exercise group and 15 in the control group). With respect to cognition, prior endurance exercise and resistance training studies show benefits on cognition at 6 or 12 months [17,35], but Chang et al [36] have shown that endurance exercise for 8 weeks leads to a statistically significant improvement in MoCA (1.6-point increase). Based on our sample size of 31, we will have 80% power with a 2-sided type I error of 0.05 to detect a significant improvement in MoCA.

### Recruitment

Participants will be recruited primarily from the UCSF movement disorders clinic. Participants will also be recruited from local PD support groups and PD events and symposiums, community neurology clinics, and self-referral from public registries (ie, ClinicalTrials.gov).

### Assignment of Interventions

#### Sequence Generation and Randomization

The principal investigator (PI) will generate the random allocation using a simple computer-generated randomization sequence.

Participants will be randomly assigned to either the exercise intervention or control group using a simple computer-generated randomization sequence with a 1:1 allocation ratio. The waitlisted control group will be offered the chance to participate in the exercise program after completing the final study visit.

#### Allocation Concealment Mechanism

The assignment sequence is not concealed from the investigator enrolling participants, and the allocation for each new participant is known to the research team. This transparent approach, though potentially susceptible to selection bias, is necessary due to the nature of the intervention and the small size of the study team. To address this limitation, an analysis of baseline characteristics will be conducted to evaluate the similarity of the 2 groups at the start of the study.

#### Implementation

After completing all baseline assessments, participants will be contacted by the investigator to receive their group assignment. Participants allocated to the exercise intervention group will be scheduled to start training with the Neuro Exercise Specialists within 2 weeks of randomization.

#### Blinding

Due to the nature of the intervention, trial participants are not blinded to their study intervention group (exercise vs waitlist control). The investigator completing the assessments is also not blinded, as the same investigator is needed to monitor safety and adherence.

### Data Collection and Management

### Plans for Assessment and Collection of Outcomes

Before the evaluations, all study participants will sign an informed consent form. The complete schedule of assessments for screening, baseline, and week 26 visits is provided in Multimedia Appendix 3. Only participants fulfilling all the inclusion and exclusion criteria at screening will be allowed to move on to baseline. Baseline visits must occur within 8 weeks of the initial screening visit. If screening tests and procedures were completed outside of this time frame, the investigator must rescreen the participant and determine if any of the screening tests and procedures need to be repeated.

Randomization will occur after all baseline assessments are completed. During the intervention period, the study team will have weekly check-in phone calls with participants to monitor adherence, AEs, and any changes in health status. Week 26 assessments will be performed at the 26-week time point ±3 weeks.

Participants taking anti-Parkinson medications will be asked to refrain from taking any anti-Parkinson medications for 12 hours prior to any scheduled assessment visit. The MDS-UPDRS parts I-IV will be completed while the participant is off medication. As soon as these assessments have been completed, the participant will be allowed to resume their medications as normal, and the remainder of the assessments will be completed while the participant is on medication. If a participant is to begin anti-Parkinson medication after enrolling in the study, an additional visit will be scheduled to administer the MDS-UPDRS parts I-IV with Hoehn and Yahr.

Participants will be assigned a unique participant identification number when they enroll in the study to maintain participant confidentiality. Data collected will be entered into the Research Electronic Data Capture (REDCap) system, a secure and HIPAA-compliant, web-based system. Any hard copies of the data will be stored securely at UCSF.

### Plans to Promote Participant Retention and Complete Follow-Up

A maximum attrition rate of 12% was accounted for in our sample size analysis. We currently have 2 participants left to randomize and have lost 2 to follow-up. Throughout the study, we have implemented multiple strategies to increase participant retention and minimize loss to follow-up. This includes (1) planning the intervention at home with virtual supervision for exercise sessions so participants do not need to frequently travel to the clinic; (2) the study team will offer additional phone calls, supervised sessions, or in-person assistance at the research center for any participants in need of support throughout the intervention; (3) video tutorials will be provided to participants, so they feel comfortable using the equipment and completing the exercises; and (4) trainers will be available to make any exercise modifications as necessary so participants can continue to complete the intervention.

### Data Management

Data collected for this study will be carefully filed and securely stored in the research offices at UCSF. Participant-identifying information, such as contact information, will be stored separately from the analysis datasets, which will include a unique study identification number for each participant. Data will be collected on case report forms that will be securely stored in locked cabinets. Deidentified data will be entered into the REDCap system, a secure and HIPAA-compliant, web-based system.

### Statistical Methods

#### Baseline Analyses

Means, SDs, medians, ranges, frequencies, and percentages will be used to describe baseline characteristics such as age, sex, education, race and ethnicity, disease duration, levodopa equivalent dose, Hoehn and Yahr stage, MDS-UPDRS motor score (part III), and MoCA. The baseline measures will be compared between the control and exercise intervention groups.

#### Statistical Methods for Primary and Secondary Outcomes

The primary outcomes are feasibility, adherence, and safety of the combined exercise program. To assess feasibility, we will estimate the overall average %HRmax for endurance training and average repetition maximum for resistance training and their corresponding 95% CIs for the exercise group at 26 weeks. We will then compare the average to the intended target intensity (82.5% for endurance exercise and 10-RM for resistance training) using a 1-sample *t* test (*α*=.05). We will use repeated measures ANOVA of weekly %HRmax and linear mixed models to assess any trends over time from weeks 7 to 26, including a random effect for time within participant as sample size permits. To measure adherence in the exercise group, the average number of days per week exercised and the duration of time (minutes) exercised at the specified intensity will be calculated. Achievement of the adherence (hypothesized to be at least 3 d/wk) will be tested using 1-sided, 1-sample Student independent group *t* tests. To assess safety, the incidence of individual AEs (including serious AEs) and exact CIs will be calculated. We will use Fisher exact or Poisson exact tests to compare exercise and control groups for AEs that are definitely, probably, or possibly related to the exercise intervention as categorical variables.

Secondary outcomes are changes from baseline in global cognitive function, motor function, and circulating biomarkers in both the exercise and control groups. Two-way repeated-measures ANOVA will be conducted to test for differences in MoCA from baseline to week 26 by group. Within each group, paired samples Student *t* tests will be conducted to test differences in MoCA from baseline to week 26. We will also examine a difference in differences analysis within and between groups overall using ANOVA. If data permit, we will use mixed-effects linear models, with time (baseline and week 26) and group (exercise and control) as the fixed effects and participant as the random effect within each model, to determine the association between change in MoCA and each of the following covariates separately to identify important differences: age, sex, education, disease duration, disease severity (defined by Hoehn and Yahr stage, MDS-UPDRS), and use of PD medications (calculated levodopa equivalent dose). Similar analysis will be performed as above to examine differences in MDS-UPDRS motor score (part III) and circulating biomarkers (neurotrophic: BDNF; inflammatory: CRP; neuroendocrine: klotho and cortisol) from baseline to week 26.

### How Missing Data Will Be Handled in the Analysis

We will handle missing data points resulting from dropouts or noncompliance using multiple imputation methods. To fully appreciate the potential influence of missing responses, sensitivity analyses will be done to examine whether the imputation method affects outcomes and whether missing data are missing-at-random.

### Methods for Additional Analyses

We will investigate several other outcomes and measures that may respond to exercise. These include participant-reported outcomes: MDS-UPDRS part II for nonmotor and motor aspects of experiences of daily living, PDQ-39 and PDAQ-15 for quality of life, PFS-16 for fatigue, and BDI-II, PAS, and AS for mood, and UCLA Loneliness Scale. We will expand cognitive analyses to evaluate changes in domain-specific functions. Using paper-based assessments (Trail Making Test and Alternating Verbal Fluency) and tablet-based assessments (NIH Toolbox), we will measure cognitive performance for each cognitive domain: executive function and working memory, processing speed and attention, visuospatial function, memory, and language. Changes from baseline in cardiorespiratory fitness (VO2peak), strength (maximal knee extension and handgrip strength), and functional capacity (6-minute walk) will also be assessed. Change in PGI-S from baseline, and Patient Global Impression of Change and Clinical Global Impression of Change will also be assessed.

Two-way repeated-measures ANOVA will be conducted to test for differences in each outcome from baseline to week 26 by group. Within each group, paired Student *t* tests will be conducted to test differences in outcome from baseline to week 26. We will use mixed-effects linear models, with time (baseline and week 26) and independent group Student *t* test (exercise and control) as the fixed effects and participant as a random effect within each model, to determine the association between change in outcome and each of the following covariates: age, sex, education, disease duration, disease severity (defined by Hoehn and Yahr stage), use of PD medications (calculated levodopa equivalent dose).

We will also collect an Exercise Attitudes, Beliefs, and Goals questionnaire at baseline and week 26. This questionnaire is designed to measure an individual’s attitudes and beliefs regarding exercise as well as confidence to continue exercising. Finally, collected blood, saliva, and CSF specimens may also be analyzed for changes in additional neurotrophic, inflammatory, and neuroendocrine biomarkers.

### Oversight and Monitoring

#### Composition of the Data Monitoring Committee, Its Role, and Reporting Structure

For this protocol, a Data Monitoring Committee is not required as this is a low-risk intervention. An Independent Safety Monitor (ISM) will oversee this clinical trial and has been approved as the sole monitor of this study by regulatory authorities. The ISM is independent of the study and has no real or apparent conflict of interest. The ISM has reviewed the research protocol and will continue to review ongoing study activities, especially data integrity, protocol adherence, and study participant safety issues at periodic intervals (approximately every 6 months). The ISM’s review will focus on AEs and reasons for follow-up losses, raising any concerns or issues with the NIH and the National Institute of Neurological Disorders and Stroke and the PI, and recommending to the National Institute of Neurological Disorders and Stroke and the PI the continuation, modification, or conclusion of the trial, while protecting the confidentiality of the trial data and the results of monitoring.

#### Frequency and Plans for Auditing Trial Conduct

Trial conduct will be reviewed by the ISM every 6 months. Annual reports including recruitment rate, impacts to the trial, deviations from the protocol, and AEs will also be prepared for submission to the funding body and the UCSF IRB.

### Protocol Amendments

Any protocol amendments will be submitted and approved by the UCSF IRB. They will also be reported to the funding agency, and the clinical trial registry will be updated.

### Dissemination Policy

The trial results will be disseminated to health care professionals and the scientific community via both publication of the results in relevant journals and presentation of the results at conferences. Results will be included in the completion report for the funding body. A summary of the results will be disseminated directly to all participants upon completion of data analysis.

## Results

The trial was approved in March 2023 and is ongoing under protocol version 1.6 (October 1, 2025). The first participant was recruited in July 2023, and the last participant was recruited in November 2025. Data collection will be completed in May 2026, with data analysis beginning immediately after. Results are expected to be published by the end of 2026 or the beginning of 2027.

## Discussion

### Principal Findings

This clinical trial is designed to evaluate the feasibility, adherence, and safety of a 26-week home-based, combined high-intensity endurance and resistance exercise intervention in PD. It is also a pilot randomized controlled trial that will assess the efficacy of the exercise intervention in improving cognition, decreasing motor symptoms, and modifying fluid-based biomarkers. The study will demonstrate whether a clinically relevant intervention that targets both the cardiorespiratory and musculoskeletal systems is safe and feasible and can be implemented at home with high adherence for individuals with PD. Given that both endurance and resistance exercise on their own have shown promise in reducing the cognitive and motor symptoms of PD [17-21], this intervention could offer a safe and feasible strategy to reduce symptom load and enhance quality of life in PD.

### Selection of Intervention

The gold-standard therapy for PD is dopaminergic medications, which reduce motor symptoms of the disease but do not slow disease progression. To date, no pharmaceutical clinical trials have been successful at slowing PD disease progression [37]. Additionally, no pharmacological therapies have been effective at improving cognitive impairments in PD, although the drug rivastigmine has shown some effectiveness in treating PD dementia but not nondementia cognitive impairments [11]. Exercise, on the other hand, has shown promise in slowing the progression of motor symptoms [19-21], as well as improving cognition in PD [17,18]. The goal of this study is to use a novel intervention and add evidence to the growing body of literature that supports exercise as a beneficial treatment for PD that has the potential to improve motor symptoms, cognition, and quality of life for those with PD.

The high-intensity endurance exercise portion of this study was modeled after the intervention used in the Study in Parkinson’s Disease of Exercise Phase II clinical trial. In this study, researchers found that 3‐4 days per week of exercising at 80%‐85% HRmax led to significantly less worsening in MDS-UPDRS motor score (part III) over 6 months compared to the moderate intensity (60%‐65% HRmax) and the usual care group [20]. We have expanded on this intervention by adding 2 days of resistance exercise per week, as endurance and resistance exercise are ideally used in tandem for exercise prescriptions in PD [28]. The EXCEL-PD intervention is being delivered at home with virtual supervision from trainers. This approach has multiple benefits, including increasing adherence as participants do not have to travel to the clinic as frequently and making the intervention more scalable, as it can be offered to participants who do not live as close to the clinic.

### Selection of Outcome Measures

In selecting the outcomes, the primary goal was to ensure that the intervention is (1) safe for participants with PD, (2) feasible to deliver at home with virtual supervision, and (3) able to achieve adequate adherence. The secondary goal of the intervention was to improve cognition, slow motor sign progression, and understand potential underlying mechanisms for any exercise-related changes by analyzing fluid-based biomarkers of the neurotrophic, inflammatory, and neuroendocrine systems. To measure cognition, we are primarily using MoCA as it is frequently used to measure global cognition. We are also using assessments for more specific cognitive domains, which will allow us to assess the effects of the exercise intervention on individual cognitive domains. Motor progression will be assessed using the MDS-UDPRS Part III (motor) score. This is the most frequently used measure of motor function in PD and will allow us to compare motor signs pre- and postintervention. We will also perform a comprehensive assessment of aerobic fitness and strength using a VO2peak test, a 6-minute walk test, and hand grip and leg extensor strength.

As fluid-based markers are increasingly being recognized for their utility as response biomarkers of exercise in PD [23], peripheral (blood and saliva) and central (CSF) biomarker measurements will allow us to examine the effects of exercise mechanistically and better understand the symptoms and pathways that exercise is acting on. We have selected key candidate biomarkers of the neurotrophic (BDNF) [38], inflammatory (CRP) [39], and neuroendocrine (cortisol and klotho) [40,41] systems as these are exercise-responsive biomarkers that could have a role in reduced PD cognitive and motor signs [23]. The outcomes chosen will also allow us to compare the results of our combined intervention to other ongoing clinical trials, such as Study in Parkinson’s Disease of Exercise Phase III, which uses some of the same outcome measures and fluid biomarkers [30]. Stored biofluid samples will allow for future analysis of additional exercise-response biomarkers that have been previously described [23].

### Recruitment and Retention

As with many clinical trials, recruitment can be a challenge for multiple reasons. A 26-week-long intervention poses a significant burden on participants, especially as this includes assessment visits in the clinic and 2 lumbar punctures to collect CSF. However, thus far, there has been ample interest in our study through the Movement Disorders clinic at UCSF, and we foresee no issues with completing recruitment. Our attrition rate thus far has been low, with only 2 control group participants electing to withdraw from the study and not returning for the final visit. Additionally, we are alleviating participant burden to the best of our abilities by using an at-home intervention and providing all necessary equipment. Our aim is that this, combined with frequent phone check-ins and trainer-supervised sessions, will keep adherence high.

### Strengths and Limitations of the Study

The main strengths of this study lie in the use of a home-based, clinically relevant combined endurance and resistance exercise intervention that targets multiple body systems. As PD progresses, it becomes increasingly difficult for those with the disease to leave the house–one major strength of this intervention is using the at-home intervention, which should greatly increase motivation and adherence. This study also collects multiple biofluids, including blood, saliva, and CSF, which significantly strengthens the study as it allows for mechanistic insights into exercise-induced changes in both the central nervous system and the periphery. Exercise intervention trials rarely collect CSF, and we will have CSF collections on all participants from 2 time points.

Limitations of this study lie largely in the duration and time points of data collection. Clinical and physiological assessments are only done pre- and poststudy, and we acknowledge that more time points for data collection and a poststudy follow-up data collection would strengthen the clinical trial. Additionally, we acknowledge that the lack of blinding in this study is a limitation and that it introduces the possibility of bias in outcome assessments. In future studies, we will have a blinded investigator complete assessments to reduce bias as much as possible. Finally, to capture the impact of disease or cognitive change on motor progression, a longer study duration may be needed. We plan to address these limitations in future clinical trials.

### Conclusions and Potential Impact of the Study

If successful, this intervention will show that a clinically relevant combined endurance and resistance exercise program is both safe and feasible and provides increased benefits to cognition and motor symptoms compared to best medical management. As there are currently no effective pharmacological treatments for cognitive impairments in PD, this study has very important clinical implications for PD treatments. We will also be able to probe the mechanisms underlying this effect using the fluid-based biomarkers collected in this trial.

## Supplementary material

10.2196/97507 Multimedia Appendix 1 Resistance exercises.

10.2196/97507 Multimedia Appendix 2 Sample endurance exercise training schedule.

10.2196/97507 Multimedia Appendix 3 Timeline of schedule of activities for the Exercise for Cognitive Excellence in Parkinson’s Disease trial.

10.2196/97507 Peer Review Report 1 Peer review report by the NST-1 - Neurological Sciences Training Initial Review Group, National Institute of Neurological Disorders and Stroke (National Institutes of Health, United States).

## Abbreviations

AE: adverse event
AS: Apathy Scale
BDI-II: Beck Depression Inventory-II
BDNF: brain-derived neurotrophic factor
CKD-EPI: Chronic Kidney Disease Epidemiology Collaboration
CRP: C-reactive protein
CSF: cerebrospinal fluid
EXCEL-PD: Exercise for Cognitive Excellence in Parkinson’s Disease
HIPAA: Health Insurance Portability and Accountability Act
HR: heart rate
HRmax: maximal heart rate
IRB: Institutional Review Board
ISM: Independent Safety Monitor
MDS-UPDRS: Movement Disorders Society Unified Parkinson’s Disease Rating Scale
MoCA: Montreal Cognitive Assessment
NIH: National Institutes of Health
PAS: Parkinson Anxiety Scale
PCP: primary care physician
PD: Parkinson disease
PDAQ-15: Parkinson’s Daily Activities Questionnaire-15
PDQ-39: Parkinson's Disease Questionnaire-39
PFS-16: Parkinson’s Disease Fatigue Scale
PI: principal investigator
REDCap: Research Electronic Data Capture
RM: repetition maximum
UCLA: University of California, Los Angeles
UCSF: University of California, San Francisco
VO2peak: maximum volume of oxygen
