# Supplementary material for: Home-Based, Virtually Supervised Combined Exercise Intervention in People With Parkinson Disease: Protocol for a Randomized Controlled Trial
Source: JMIR Res Protoc. 2026 Jun 30;15:e97507. doi: 10.2196/97507 (PMC13317673; doi:10.2196/97507)
Supplement: Multimedia Appendix 1 [file resprot-v15-e97507-s001.pdf]

Supplementary Table 1 Resistance exercises

**Weeks 1-13**			
Circuit 1a	Squat with Dumbbell to Overhead Press	Chest Press	Dumbbell Bent Over Row
Circuit 1b	Plank	Side Lunge with Lateral Dumbbell Rise	Kneeling Dumbbell Diagonal Chop
**Weeks 14-26**			
Circuit 2a	Squat/Sit to Stand Dumbbell Diagonal Woodchop	Lateral Lunge/Step Dumbbell Around the World	Bird Dog
Circuit 2b	Push Up (modified or regular)	Dumbbell – Prone Reverse Fly	Reverse Lunge with Dumbbell
