# Supplementary material for: Home-Based, Virtually Supervised Combined Exercise Intervention in People With Parkinson Disease: Protocol for a Randomized Controlled Trial
Source: JMIR Res Protoc. 2026 Jun 30;15:e97507. doi: 10.2196/97507 (PMC13317673; doi:10.2196/97507)
Supplement: Multimedia Appendix 2 [file resprot-v15-e97507-s002.pdf]

Supplementary Table 2 Sample endurance exercise training schedule

	Description of Session	Supervised or Unsupervised
**Week 1-2 Familiarization**		
Day 1	Familiarization with treadmill	Supervised
Day 2	Familiarization with treadmill/heart rate monitor	Supervised
Day 3	Familiarization with weights	Supervised
Day 4	Familiarization with weights	Supervised
Day 5	Familiarization with movements for resistance training	Supervised
Day 6	Familiarization with movements for resistance training	Supervised
**Week 3 Ramp Up**		
Day 1	Treadmill - 25 min +10 min warm up/cool down, 60-65% max HR	Supervised
Day 2	Resistance training 20 min + 10 min warm up/cool down	Supervised
Day 3	Treadmill - 25 min +10 min warm up/cool down, 60-65% max HR	Supervised
**Week 4 Ramp Up**		
Day 1	Treadmill - 30 min +10 min warm up/cool down, 65-70% max HR	Supervised
Day 2	Resistance training 25 min + 10 min warm up/cool down	Supervised
Day 3	Treadmill - 30 min +10 min warm up/cool down, 65-70% max HR	Supervised
**Week 5 Ramp Up**		
Day 1	Treadmill - 30 min +10 min warm up/cool down, 70-75% max HR	Supervised
Day 2	Resistance training 30 min + 10 min warm up/cool down	Supervised
Day 3	Treadmill - 30 min +10 min warm up/cool down, 70-75% max HR	Unsupervised
Day 4	Resistance training 30 min + 10 min warm up/cool down	Unsupervised
**Week 6 Ramp Up**		
Day 1	Treadmill - 40 min +10 min warm up/cool down, 80-85% max HR	Supervised
Day 2	Resistance training 40 min + 10 min warm up/cool down	Supervised
Day 3	Treadmill - 40 min +10 min warm up/cool down, 80-85% max HR	Unsupervised
Day 4	Resistance training 40 min + 10 min warm up/cool down	Unsupervised
Day 5	Treadmill - 40 min +10 min warm up/cool down, 80-85% max HR	Unsupervised
**Weeks 7-26**		
Day 1	Treadmill - 40 min +10 min warm up/cool down, 80-85% max HR	Supervised 1 time per month, otherwise unsupervised
Day 2	Resistance training 40 min + 10 min warm up/cool down	Supervised 3 times per month, otherwise unsupervised
Day 3	Treadmill - 40 min +10 min warm up/cool down, 80-85% max HR	Unsupervised
Day 4	Resistance training 40 min + 10 min warm up/cool down	Unsupervised
Day 5	Treadmill - 40 min +10 min warm up/cool down, 80-85% max HR	Unsupervised
