# Supplementary material for: Home-Based, Virtually Supervised Combined Exercise Intervention in People With Parkinson Disease: Protocol for a Randomized Controlled Trial
Source: JMIR Res Protoc. 2026 Jun 30;15:e97507. doi: 10.2196/97507 (PMC13317673; doi:10.2196/97507)
Supplement: Multimedia Appendix 3 [file resprot-v15-e97507-s003.docx]

**Table**

|  | **Study period** | | | | |
| --- | --- | --- | --- | --- | --- |
| **Time point** | Phone prescreen | In-person screening | In-person baseline | Weekly phone calls | In-person week 26 |
| **Pre-enrollment** |  |  |  |  |  |
| Prescreen | X |  |  |  |  |
| **Enrollment and Screening procedures** |  |  |  |  |  |
| Informed consent |  | X |  |  |  |
| Demographics |  | X |  |  |  |
| Review of medical history |  | X |  |  |  |
| Review of PD history |  | X |  |  |  |
| Medication log |  | X | X |  | X |
| Physical and neurological exam |  | X |  |  | X |
| Blood draw for exercise clearance |  | X |  |  |  |
| Inclusion and exclusion criteria review |  | X | X |  | X |
| **Biofluid collections** |  |  |  |  |  |
| Blood draw for biomarkers |  |  | X |  | X |
| Lumbar puncture for CSF biomarkers |  |  | X |  | X |
| Saliva sample collection for biomarkers |  |  | X |  | X |
| **Assessments** |  |  |  |  |  |
| Fall questionnaire |  | X |  |  |  |
| MoCA |  | X |  |  | X |
| BDI-II |  | X |  |  | X |
| MDS-UPDRS I-IV |  |  | X |  | X |
| 6-minute walk |  |  | X |  | X |
| Strength testing |  |  | X |  | X |
| VO2peak |  |  | X |  | X |
| PDQ-39 |  |  | X |  | X |
| PDAQ-15 |  |  | X |  | X |
| PFS-16 |  |  | X |  | X |
| PAS |  |  | X |  | X |
| AS |  |  | X |  | X |
| Loneliness Scale |  |  | X |  | X |
| Exercise Attitudes, Beliefs, and Goals Survey |  |  | X |  | X |
| Cognitive battery |  |  | X |  | X |
| PGI-S |  |  | X |  | X |
| PGI-C |  |  |  |  | X |
| CGI-C |  |  |  |  | X |
| **Interventions and adherence** |  |  |  |  |  |
| Exercise intervention |  |  |  | | |
| Control intervention |  |  |  | | |
| Session supervision |  |  |  | | |
| Adherence and heart rate data verification |  |  |  | | |
| Adverse events reporting |  |  | X X | | |

Abbreviations: PD (Parkinson's disease); MoCA (Montreal Cognitive Assessment); BDI-II (Beck Depression Inventory, second edition); CSF (cerebrospinal fluid); MDS-UPDRS I-IV (Movement Disorders Society - Unified Parkinson's Disease Rating Scale, part I-IV); VO2peak (peak oxygen uptake); PDQ-39 (The Parkinson's Disease Questionnaire-39); PDAQ-15 (Penn’s Parkinson’s Daily Activities Questionnaire-15); PFS-16 (Parkinson’s Disease Fatigue Scale); PAS (Parkinson Anxiety Scale); AS (Apathy Scale); PGI-S (Patient Global Impression of Severity); PGI-C (Patient Global Impression of Change); CGI-C (Clinician Global Impression of Change).
