# Supplementary material for: Home-Based, Virtually Supervised Combined Exercise Intervention in People With Parkinson Disease: Protocol for a Randomized Controlled Trial
Source: JMIR Res Protoc. 2026 Jun 30;15:e97507. doi: 10.2196/97507 (PMC13317673; doi:10.2196/97507)
Supplement: Peer Review Report 1 [file resprot-v15-e97507-s004.pdf]

SUMMARY STATEMENT
(Privileged Communication)

PROGRAM CONTACT:
Beth-Anne Sieber
301-496-5680
sieberb@ninds.nih.gov

Release Date: 03/15/2022
Revised Date:

Application Number: 1 K23 NS123506-01A1
Formerly: 1K23NS123506-01

Principal Investigator

LUTHRA, NIJEE

Applicant Organization: UNIVERSITY OF CALIFORNIA, SAN FRANCISCO

Review Group: NST-1
NST-1 Study Section
NST-1

Meeting Date: 01/31/2022
Council: MAY 2022
Requested Start: 07/01/2022
RFA/PA: PA20-206
PCC: SIEBEBND

Project Title: Enhancing Cognitive Resilience In Parkinson's Disease: Role Of Antiaging Protein Klotho
SRG Action: Impact Score:31
Next Steps: Visit https://grants.nih.gov/grants/next_steps.htm
Human Subjects: 30-Human subjects involved - Certified, no SRG concerns
Animal Subjects: 10-No live vertebrate animals involved for competing appl.
Gender: 1A-Both genders, scientifically acceptable
Minority: 1A-Minorities and non-minorities, scientifically acceptable
Age: 3A-No children included, scientifically acceptable

Project Year	Direct Costs Requested	Estimated Total Cost
1	176,400	190,272
2	176,400	190,272
3	176,400	190,272
4	176,400	190,272
5	176,400	190,272
TOTAL	882,000	951,360

ADMINISTRATIVE BUDGET NOTE: The budget shown is the requested budget and has not been adjusted to reflect any recommendations made by reviewers. If an award is planned, the costs will be calculated by Institute grants management staff based on the recommendations outlined below in the COMMITTEE BUDGET RECOMMENDATIONS section.

1K23NS123506-01A1 Luthra, Nijee

RESUME AND SUMMARY OF DISCUSSION: This is a resubmitted K23 application from Nijee Luthra, M.D., Ph.D., who is an Assistant Clinical Professor in Neurology at the University of California, San Francisco (UCSF). Reviewers indicated Dr. Luthra to be an outstanding candidate for a K23. They indicated she has a strong research background with a Ph.D. in Neuroscience and is well-published. Dr. Luthra has a team of outstanding mentors and advisors and is mentored at UCSF by Dr. Caroline Tanner, and co-mentored by Drs. Isabel Allen (UCSF), Kathleen Poston (Stanford) and Daniel Cross (Northwestern). Drs. Dena Dubal (UCSF) and Daniel Weintraub (U. Pennsylvania) are scientific advisors for the proposed research. Reviewers indicated this was a strong application in its first submission and is now even better given the career development plan (CDP) is now stronger, more thorough and appropriate. The CDP now involves a change of the coursework with DAT image scan progression studies included and with Dr. Poston now as a mentor and better statistical advice with the addition of a statistician as a co-mentor. Overall, the CDP now has structured meetings added and is now focused on the design, conduct, and analysis of randomized clinical trials, advanced quantitative research methods including training in epidemiology and advanced biostatistics, analysis and interpretation of multimodal biomarkers, participation in meetings with the SPARX3 core imaging group, and gaining a better understanding of cognitive change and cognitive assessments in patients with Parkinson's disease (PD). Minor concern with the CDP related to the time a 15-course program will involve. The quality of the mentors, the CDP and the research training plan will likely lead to independence and competitiveness for a future R01. Institutional commitment was considered to be excellent.

The proposed research training is based upon recent findings in PD regarding the longevity hormone, klotho, which is an aging regulator protein that increases with exercise and decreases with aging and PD. It also was recently found that higher levels of klotho protein are present in those carrying one allele of the KLOTHO gene variant KL-VS. Dr. Luthra proposes to study whether KL-VS heterozygous status is associated with a slower rate of decline in global cognitive function and particularly in executive function as well as with a reduced decline in dopaminergic denervation (as based upon DAT scans), and/or with a lower burden of CSF amyloid-ß 1-42, tau, and α-synuclein. Dr. Luthra also proposes to determine whether klotho levels increase in response to a home exercise program in patients with PD and whether klotho levels correlate with improved cognitive abilities in these patients, among other measures. Reviewers indicated the proposed research is sound and elegant, uses a mixed effects multiple regression model for correcting for age, sex, education, disease severity, and levodopa equivalent dose, with vitamin D supplementation as a covariate, and is backed by strong preliminary data. The research is highly important to the field and one of its most important goals is to test feasibility, establish safety, adherence, and efficacy of the home-based exercise intervention. Despite the strengths, multiple minor weaknesses were noted by some but not all of the reviewers. Prime among these were that the number and type of outcome measures may be unnecessarily burdensome to some of the participants and while the study is described as a feasibility study, effect-based statistics are being proposed. Concerns also included that dopamine transporter function could be affected by the exercise program, that it was unclear how the cognitive data will be harmonized from different sources in the retrospective Aim 1, and whether it is reasonable to ask people in the control group not to exercise at all for 16 weeks. This last concern was qualified that Dr. Luthra does not state they cannot do any exercise at all, but that the controls will be instructed not to change their physical activity habits for 16 weeks. Moreover, they will be given the opportunity to participate in the home-based exercise protocol after the 16 weeks should they wish. Overall, reviewers cited numerous strengths to this candidate, the proposed training and the research plan which should set Dr. Luthra up nicely for being competitive and for moving into independence. All noted weaknesses/concerns were considered minor.

Responsible Conduct of Research (RCR): Acceptable

DESCRIPTION (provided by applicant): This is an application for a Mentored Patient-Oriented Research Career Development Award (K23) by Dr. Nijee Luthra, a neurologist specializing in movement disorders. As the number of people with Parkinson's disease (PD) is expected to grow to pandemic proportions, cognitive deterioration associated with PD will prevail in a majority of this population. The integrated career development and research plan proposed for this K23 award is motivated by an increasing awareness of the urgent need to identify pathways that target cognitive impairment in PD. Exercise has potent anti-aging effects and can influence cognitive outcomes in PD but its underlying mechanisms remain unclear. Identifying biomarkers that respond to exercise, and how they associate with cognition and underlying disease pathology may elucidate key mechanisms for countering cognitive decline. This proposal focuses on longevity hormone klotho as a biomarker that may mediate exercise-induced benefits and contribute to cognitive resilience. Klotho has been shown to enhance cognition, synaptic plasticity, and brain volumes in mouse models of aging and neurodegeneration. Dr. Luthra and her team have also recently shown that klotho associates with better cognitive function in PD patients. While klotho levels decrease with aging and in PD, physical exercise robustly increases klotho levels in healthy adults. Whether exercise increases klotho in PD and protects against cognitive decline is not yet known. To this end, the aims of this research proposal are: 1) knowing its positive link with cognition, to now investigate whether klotho modifies underlying markers of PD pathology, and 2) in parallel, design a pilot study to determine if klotho increases in response to exercise in PD and correlates with improved cognitive abilities. Answers to these questions would highlight the potential of klotho as an exercise-related biomarker and as a new pathway for targeting cognitive dysfunction. The research plan is complemented by didactic and practical training in design and conduct of clinical trials, advanced biostatistical methods, and analysis and interpretation of multimodal biomarkers and cognitive assessments. To accomplish the goals of this research and training, the candidate has assembled a mentoring team with decades of experience in clinical trials and exercise interventions, along with expertise in biomarkers and cognitive impairment in PD. Completion of this research plan and training objectives will allow the candidate to advance her career in becoming an independent clinical investigator focused on investigating strategies such as exercise and multiple related biomarkers that mediate cognitive resilience in PD.

PUBLIC HEALTH RELEVANCE: Cognitive impairment begins early in Parkinson's disease (PD) and progresses to dementia in a majority of patients, impairing quality of life and contributing to growing health-related costs. The proposed study is designed to study exercise and a novel aging-regulator, klotho, that may mediate-exercise induced benefits on cognition. This research has the potential to fill a major gap in identifying novel therapeutic strategies to counter cognitive decline in PD.

CRITIQUES: *Please note that the evaluations and criterion scores from individual reviewers are provided below in an essentially unedited form. These were prepared prior to the review meeting and may not have been updated or revised after the discussion at the meeting. Therefore, they may not fully reflect the final opinions of the individual reviewers at the close of group discussion or the final majority opinion of the group. The Resume and Summary of Discussion above summarizes the outcome of the group discussion.*

CRITIQUE 1
Candidate: 2
Career Development Plan/Career Goals /Plan to Provide Mentoring: 2
Research Plan: 2
Mentor(s), Co-Mentor(s), Consultant(s), Collaborator(s): 2
Environment and Institutional Commitment to the Candidate: 2

Overall Impact: This is a well-written revised application from an outstanding candidate, a Neurologist with a PhD in neuroscience, with a very strong research background and publishing track record. The career development plan is now stronger with the addition of a statistician; change of the coursework in imaging now focused on DAT scan progression studies with Dr. Poston as mentor and work with the Core for the ongoing SPARX3 trial; and addition of structured meetings. In response to critiques suggesting *a lack of investigator-initiated research grants or pilot funding; prior training unclear,* the candidate shows intramural research support and from various clinical trials. The mentors are outstanding and clearly participated in a very constructive way to address the criticisms and to produce the revised application. The research proposal has been modified addressing all the critiques with the addition of preliminary data and in the study design with the addition of different measurements and a control group for Aim 2. Indeed, as stated by the candidate, the proposed studies will serve as "*a model … to develop the research and analytical skills [that can be applied] … to other biomarkers*". The revised research proposal is strong with promising preliminary data. Undoubtably, it will provide a good training ground adequate to the candidate expertise level, it will likely lead to R01 and to the candidate independence. The institutional commitment is excellent.

1. Candidate

Strengths

- The candidate, a Neurologist with PhD in Neuroscience (research on alpha-synuclein) has obtained a position of Assistant Clinical Professor of Neurology at UCSF following a movement disorder fellowship in 2017. The candidate has a solid research experience, as she participated in two observational longitudinal studies and eight clinical trials in different phases. In particular, Dr. Luthra has been a site co-PI or co-investigator in industry- or foundation-funded clinical trials: investigator for PPMI (data that will be used for this research proposal); co-PI for NIH/NINDS funded phase III trial of exercise in PD (SPARX3) and the proposed use of exercise as means of increasing klotho in PD (highly relevant for this proposal); Biogen Biotechnologies (Wearable Assessments in the Clinic and Home in PD, WATCH-PD, relevant for this proposal). A PubMed search shows eighteen papers (two more since the last application) with first authorship in eight of them, all somewhat related to the proposed research plan. All this while going through two maternity leaves. The recommendation letters are outstanding.
- Altogether, this seems an outstanding candidate with an excellent background in research publication and direct participation to projects that suggest exceptional and continuous commitment to research in PD.

Weaknesses

- No weaknesses.

2. Career Development Plan/Career Goals and Objectives

Strengths

- The areas of development listed by the candidate are: (***1***) design, conduct, and analysis of RCTs [courses to *acquire certification of Advanced Training in Clinical Research at UCSF + NINDS Clinical Trials Methodology Course CTMC* and direct mentorship with primary mentor, Dr. Caroline Tanner and co-mentor Dr. Corcos]; (***2***) advanced quantitative research methods [training in epidemiology and advanced biostatistics *provided by Advanced Training in Clinical Research at UCSF* and direct mentorship with co-mentor Dr. Allen in monthly meetings]; ***3***) analysis and interpretation of multimodal biomarkers [direct mentorship with co-mentor Dr. Poston in monthly meetings, participation in meetings in particular with SPARX3 core imaging group to analyze 370 scans], and (4) understanding of cognitive changes/assessments in PD [direct mentorship with co-mentor Dr. Poston in virtual meetings and personal meeting with Dr. Dubal].
- This is an excellent, well-designed and feasible training plan with courses, hands on work and direct mentorship. This training plan starts from a solid background and will provide new training and fill in some gaps. Benchmarks of success well-structured and feasible.
- The clinical trial experience described for Aim 2 will provide an important contribution to the applicant's research career development for the application of the knowledge acquired with courses about design, conduct, and analysis of RCTs as well as epidemiology and advanced biostatistics.

Weaknesses

- No weakness noted

3. Research Plan

Strengths

- The central hypothesis is based on recent findings in PD about the longevity hormone, klotho, an aging regulator protein that increases with exercise and decreases with aging and PD. Higher levels of klotho protein result from carrying one allele of the KLOTHO gene (variant KL-VS). The candidate proposes two aims to answer to questions. The first question is: *Is KL-VS heterozygous status associated with slower rate of decline in global cognitive function and particularly in executive function; with reduced decline in dopaminergic denervation with DAT scans; with lower burden of CSF amyloid-beta 1-42, tau, a-synuclein*? This question is addressed with a retrospective longitudinal study of data bases (AMP- PD and PPMI, about 1,300 patients followed for 3 or 5 years).
- The candidate proposes a sound and elegant approach that starts with mixed effects multiple regression models correcting for age, sex, education, disease severity, and levodopa equivalent dose, with vitamin D supplementation as a covariate.
- The second question is: *Do klotho levels increase in response to a home exercise program in PD and do they correlate with improved cognitive abilities and other measurements?* This will be addressed with a prospective pilot study that includes a group of patients home-based exercise program (16 weeks, different exercise components) and a No exercise group (or *wait-list group*) that will be instructed not to change their physical activity habits for 16 weeks, will be put in a waiting list and will have the option to participate in the home-based protocol after 16 weeks.
- One of the most important goals is to test feasibility, establish safety, adherence, and efficacy of this intervention. Increases of blood and CSF klotho levels, changes in cognition and physical performance as well as possible correlation between performance and biochemical measurements will be assessed.

- There are preliminary data directly supporting the background, the feasibility and the possible outcome of the proposed studies. In particular, the candidate mentions published data about the fact that KL-s heterozygotes have higher klotho blood levels in normal subjects (data from the literature), depending on age (Dr. Dubal's data), PD status and exercise effects on klotho in normal subjects. Also, there are preliminary non-published data coming from two independent data sets (N=355 and N=83) showing better executive performance in the KL-VS carriers. In addition, the candidate presents a similar frequency of KL-VS allele in normal and PD population (data extracted from PPMI). Finally, data published by the co-mentor, Dr. Corcos and colleagues, shows that high-intensity treadmill exercise may be feasible in patients with PD.
- This is a good design with two different types of studies, each standing independently, with multimodal approach. This novel study may have great clinical significance also providing an excellent training ground for the skills described in the development plan. Importantly, there will be material for R01.

Weaknesses

- There are no weaknesses or concerns.

4. Mentor(s), Co-Mentor(s), Consultant(s), Collaborator(s)

Strengths

- The Mentoring team is superb, as it is composed by experts in their field all well-funded. Dr. Tanner, a highly recognized movement disorder expert, is well posed to be the primary mentor, with a lot of expertise in training physician scientists.
- Dr. Dubal an UCSF Endowed Chair in Aging and Neurodegenerative Disease with research focused on mechanisms of resilience in aging and neurodegenerative disease and klotho can provide specific guide on klotho.
- Dr. Poston, chief of movement disorders and expert on imaging and biological biomarkers associated with the cognitive, motor, and behavioral symptoms of synucleinopathies, will help in the imaging part.
- The addition of Dr. Allen a professor of biostatistics reinforces the local team of co-mentors.
- Additional co-mentors outside UCSF, will be Dr. Corcos, PI of SPARX3, and Dr. Weintraub, a renowned expert in psychiatric and cognitive complications of PD.
- The involvement of the mentors is outstanding, as shown by their participation to the response to the previous review in their support letters.

Weaknesses

- No weaknesses noted.

5. Environment:

Strengths

- The Chair assures that the candidate will devote at least 75% (at least nine person months) for research and career development activities with the remaining 25% dedicated to clinical work (one full day per week for movement disorders outpatient care, 20%) and teaching (one half day of every 2 weeks, i.e., 5%) medical students, residents, and fellows.
- The candidate will have no in-patient or on-call responsibilities.

Weaknesses

- No weaknesses noted.

Study Timeline:

Strengths

- The study timeline has been described in detail, with start-up activities, rate of enrollment and follow-up assessments. The candidate also includes strategies to maximize rate of enrolment, including tapping into the SPARX-3 population.

Weaknesses

- None

Protections for Human Subjects: Acceptable Risks and Adequate Protections

- All the risks have been appropriately acknowledged and discussed including protection against them. Benefits of the study as well as the importance of the knowledge to be gained have been properly described. Most importantly, the control group for Aim 2 (No exercise group) will be instructed not to change their physical activity habits for 16 weeks, will be put in a wait-list control group and will have the option to participate in the home-based protocol. This approach eases all ethical concerns for this reviewer.

Data and Safety Monitoring Plan (Applicable for Clinical Trials Only): Acceptable

- Appropriate data and safe monitoring plan has been described.

Inclusion of Women, Minorities, and Ages Across the Lifespan:

- Sex/Gender: G1A: Both sexes used: Scientifically Acceptable
- Race/Ethnicity: M1A: Minority and non-minority: Scientifically Acceptable
- Inclusion/Exclusion Across the Lifespan: C3A: No Children Included (Age ≥ 18); Scientifically Acceptable
    - PD is a disease that usually affects adults.

Vertebrate Animals: Not applicable (No Vertebrate Animals)

Biohazards: Not applicable (No Biohazards)

Resubmission: Acceptable

- The candidate has successfully responded to all criticisms of the previous review.

Training in the Responsible Conduct of Research: Acceptable

Comments on Format (Required):

- Format has been specified and is adequate

Comments on Subject Matter (Required):

- Subject matter has been specified and is adequate

Comments on Faculty Participation (Required):

- Faculty participation has been specified and is adequate

Comments on Duration (Required):

- Duration of instruction has been specified and is adequate

Comments on Frequency (Required):

- Frequency of instruction has been specified and is adequate

Authentication of Key Biological and/or Chemical Resources: Not applicable (No Relevant Resources)

Select Agent Research: Not applicable (No Select Agents)

Resource Sharing Plans Not applicable (No Relevant Resources)

Budget and Period of Support: Recommend as Requested

CRITIQUE 2
Candidate: 2
Career Development Plan/Career Goals /Plan to Provide Mentoring: 1
Research Plan: 3
Mentor(s), Co-Mentor(s), Consultant(s), Collaborator(s): 2
Environment and Institutional Commitment to the Candidate: 1

Overall Impact: This is a revised K23 application by Nijee Luthra at the University of California San Francisco on Enhancing Cognitive Resilience In Parkinson's Disease: Role Of Antiaging Protein Klotho. The revised application response is thorough and clear. The career development plan, mentors, and research strategy will support the candidate in her goal of an independent research career that focuses on strategies such as exercise and multiple related biomarkers that mediate cognitive resilience in PD. Minor weakness in the feasibility part of AIM are found. Enthusiasm is high.

1. Candidate

Strengths

- The candidate outlines a history of training and previous research experiences that have led to this point and how a K award will support the development of an independent investigator. The personal narrative in the biosketch is particularly strong.
- The candidate has 17 publications on MyNCBI, 8 as first author. Most in Parkinson’s Disease.
- PhD in neuroscience, Assistant professor
- The candidate has experience in longitudinal research design, and Co-PI for trial on effect of exercise in PD which is relevant to this proposal

Weaknesses

- None noted

2. Career Development Plan/Career Goals and Objectives

Strengths

- Table 1 is exemplary
- The combination of training in clinical trials, advanced quantitative methods, analysis and interpretation of biomarkers, cognitive impairment in PD, and other actives are appropriate and strong for content, scope, phasing, and duration.
- Benchmarks for success are reported
- Letters from mentors are specific and add confidence and consistency to the career development plan

Weaknesses

- None reported

3. Research Plan

Strengths

- There is a strong scientific premise for the possibility that klotho
- This study utilizes the Accelerating Medicines Partnership in PD (AMP-PD) and Parkinson's Progression Marker's Initiative (PPMI) databases for AIM 1
- This study uses a pilot RCT with a waitlist control for AIM 2
- The feasibility study considers adherence to the exercise and is sufficiently detailed and comprehensive
- Preliminary data is expanded

Weaknesses

- The number and type of outcome measures maybe unnecessarily burdensome to participants
- The study is described as feasibility and effect-based statistics are proposed

4. Mentor(s), Co-Mentor(s), Consultant(s), Collaborator(s)

Strengths

- The mentors have considerable expertise that matches the goals the candidate lists

Weaknesses

- None noted

5. Environment:

Strengths

- The UCSF's neurology and neurosurgery clinical departments and the combined UCSF Institute for Neurosciences for clinical and research facilities support this work and the candidate.

Weaknesses

- None noted

Protections for Human Subjects: Unacceptable Risks and/or Inadequate Protections

- Consider recording the exercise for the waitlist control group instead of withholding exercise during this time.

Data and Safety Monitoring Plan (Applicable for Clinical Trials Only): Acceptable

Inclusion of Women, Minorities, and Ages Across the Lifespan:

- Sex/Gender: G1A: Both sexes used: Scientifically Acceptable
- Race/Ethnicity: M1A: Minority and non-minority: Scientifically Acceptable

- For NIH-Defined Phase III trials, Plans for valid design and analysis: Not applicable
- Inclusion/Exclusion Across the Lifespan: C3A: No Children Included (Age ≥ 18); Scientifically Acceptable

Vertebrate Animals: Not applicable (No Vertebrate Animals)

Biohazards: Not applicable (No Biohazards)

Resubmission*:* Acceptable

Training in the Responsible Conduct of Research: Acceptable

Authentication of Key Biological and/or Chemical Resources: Not applicable (No Relevant Resources)

Select Agent Research: Not applicable (No Select Agents)

Resource Sharing Plans Not applicable (No Relevant Resources)

Budget and Period of Support: Recommend as Requested

CRITIQUE 3
Candidate: 3
Career Development Plan/Career Goals /Plan to Provide Mentoring: 3
Research Plan: 3
Mentor(s), Co-Mentor(s), Consultant(s), Collaborator(s): 2
Environment and Institutional Commitment to the Candidate: 2

Overall Impact: This is a resubmission application that has been strengthened substantially. The applicants is a well-trained MD-PhD applicant who has a very good publication record from graduate school and earlier work, but who has only two first author publications since completion of fellowship in 2017. The applicant presents interesting and innovative ideas about klotho and relationship to cognitive decline in PD. The design and methods have been strengthened substantially include a robust statistical plan. There remain some concerns about how this project will prepare the applicant for independence and whether the institution is committed to her long-term success.

1. Candidate

Strengths

- Excellent training in neurology and movement disorders
- Very good research potential
- Local leadership role in multiple multi-site clinical trials

Weaknesses

- Little investigator-initiated research aside from database or medical record analysis

2. Career Development Plan/Career Goals and Objectives

Strengths

- Plan for advanced training in clinical research
- Planned participation in the Clinical Trials Methods course

- Strong plan for education in design and statistics, now with the addition of formal mentorship by a biostatistician
- Very good timeline clearly integrated with research plan.

Weaknesses

- How will the career development plan prepare the applicant for a career as an independent investigator? This remains unclear.
- There is a plan for biannual meetings with her mentor team, but it is not specified how progress will be assessed and how the plan may be modified based on that assessment.

3. Research Plan

Strengths

- Innovative concept and important problem
- Leveraging large existing databases for aim 1
- The research plan is directly relevant to the applicant's development interests
- The statistical analysis plan is clearly stated, including power analyses
- Preliminary data are clearly presented and the plan for longitudinal analysis is stated clearly.
- Inclusion of a waiting-list control group reduces some confounds related to exercise.
- Inclusion of dopamine transporter activity (DATSCANS) is a potential strength.
- While a strength, the dopamine transporter activity may be difficult to interpret in a rigorous manner. DAT is regulated in a complex manner. If there are differences in DAT activity based on klotho genotype and also in relation to PD severity, how relationships among these variables be addressed.

Weaknesses

- The applicant specifies the cognitive tests used in each cohort, but there are concerns about the comparability across these cohorts. More information on associations between the individual cognitive tests and the broader MOCA and MMSE is needed.

4. Mentor(s), Co-Mentor(s), Consultant(s), Collaborator(s)

Strengths

- The mentor team is very strong and represents broad and deep expertise.
- The inclusion of a biostatistician on the mentoring team is a strength.

Weaknesses

- The plan for monitoring and evaluating progress is stated by the applicant, but there is no confirmation by the mentors.
- Existing support for the proposed project is uncertain. How will research costs beyond what is supported by the K23 be supported?

5. Environment:

Strengths

- The environment is outstanding

- There is a letter of support from the Department Chair that is strong.

Weaknesses

- The Chair's letter does not specify commitment to the candidate if this application is not funded.

Protections for Human Subjects: Acceptable Risks and Adequate Protections

Data and Safety Monitoring Plan (Applicable for Clinical Trials Only): Acceptable

Inclusion of Women, Minorities, and Ages Across the Lifespan:

- Sex/Gender: G1A: Both sexes used: Scientifically Acceptable
- Race/Ethnicity: M1A: Minority and non-minority: Scientifically Acceptable
- For NIH-Defined Phase III trials, Plans for valid design and analysis:
- Inclusion/Exclusion Across the Lifespan: C3A: No Children Included (Age ≥ 18); Scientifically Acceptable
    - No concerns

Vertebrate Animals: Not applicable (No Vertebrate Animals)

Biohazards: Not applicable (No Biohazards)

Resubmission*:* Acceptable

Training in the Responsible Conduct of Research: Acceptable

Comments on Format (Required):

- Acceptable

Comments on Subject Matter (Required):

- Acceptable

Comments on Faculty Participation (Required):

- Acceptable

Comments on Duration (Required):

- Acceptable

Comments on Frequency (Required):

Authentication of Key Biological and/or Chemical Resources: Not applicable (No Relevant Resources)

Select Agent Research: Not applicable (No Select Agents)

Resource Sharing Plans Acceptable

Budget and Period of Support: Recommend as Requested

CRITIQUE 4
Candidate: 2
Career Development Plan/Career Goals /Plan to Provide Mentoring: 3
Research Plan: 4

Mentor(s), Co-Mentor(s), Consultant(s), Collaborator(s): 2
Environment and Institutional Commitment to the Candidate: 2

Overall Impact: This is a revised K23 application from a well-trained neurologist. The application details the updated plan to determine how klotho longitudinally associates with cognitive decline and underlying neural substrates of PD (Aim 1) and new plans to conduct a randomized pilot study to evaluate feasibility of a home-based exercise intervention and measure effects on circulating klotho and cognition (Aim 2). The application was revised under the guidance of a biostatistician. The applicant has addressed some of the statistical concerns noted in the original application, however, the revision has multiple minor weaknesses in the statistical plans.

1. Candidate:

Strengths

- Outstanding candidate

Weaknesses

- *Reviewer did not provide any comments regarding weaknesses*

2. Career Development Plan/Career Goals & Objectives/Plan to Provide Mentoring:

Strengths

- Training in “advanced quantitative methods” is emphasized.
- Statistical computing didactic is an important addition to the CDP.

Weaknesses

- The “Advanced Training in Clinical Research Certificate Program” is now described as a 15-course program. It is not clear how the many didactics in epidemiology and biostatistics (3 course-series in epi; 4 course series in biostats) align with the proposed work or career development plans.

3. Research Plan:

Strengths

- The research plan addresses an important unmet need.
- The applicant has access to large longitudinal data for several PD cohorts.

Weaknesses

- The presentation of the preliminary data is often missing the number of observations (in fact, Table 3 is labeled as “Frequencies” but there are no counts presented).
- Strength of conclusions drawn throughout the background sections are not well-supported. In 4.C-1.3: that KL-VS homozygosity decreases circulating klotho appears to be based on n=5. It is also unclear whether the observed increase in circulating klotho in heterozygotes has been replicated.
- Building on the previous point, based on the information presented, the relationship between klotho and KL-VS does not seem to be well-established. In Aim 1, there is no confirmation that klotho is indeed increased “as a function of KL-VS”. Instead, in each of the hypotheses of Aim 1,

the applicant is proposing to assess the relationship between KL-VS heterozygosity and cognitive decline/dopaminergic denervation/CSF abnormalities, and is never actually assessing "increased klotho".

- Aim 1: How will rates be compared (e.g., slope of change)?
- Aim 1: the Data Analysis section for H1a describes that each cognitive test will be modeled separately (and that FDR method will be used to adjust for multiple comparisons). Do all analyses need to be significant (after correction for multiple comparisons) in order to state that there is a slower rate of cognitive decline in the heterozygous group or is there a (undefined) composite outcome?
- Aim 1: in the H1A statistical power section, the "significant difference" to be detected is not well defined or justified. Are cross-sectional mean differences observed in healthy individuals (i.e., Cohen's d for global/executive function) the benchmark for significance in differences in rates of cognitive decline in PD?
- Aim 1: in H1b, power of 90% appears to be presented for the 0.38 effect size. The power to detect an effect size of 0.3 is about ~73% with provided Ns and alpha.
- Aim 2: the assumed standard deviation for the sample size calculation is not provided (nor justified). The same is true for H1B.
- Aim 2: the sample size inflation factor for 20% attrition should be at least 1/(1-.20). (The applicant may also want to consider the impact of "cross-over" in this design when selecting an inflation factor for LTFU/cross-over [i.e., if subjects randomized to exercise group do not exercise the effect may be attenuated]).
- Aim 2: the choice to use average number of days of exercise as the outcome in H1a is not justified.
- Aim 2: the rationale for proposing non-inferiority tests is not clear and this is an atypical application (without a clear non-inferiority margin). The test described for H2a actually appears to be a one-sided superiority test.
- Aim 2: it is not clear what is being presented for the power calculation in H2b: within group change (exercise group only) or between group difference? Either way, this reviewer is unable to independently replicate power calculation. In order to justify randomization to control, the comparison between groups should be clearly stated and justified.

4. Mentor(s), Co-Mentor(s), Consultant(s), Collaborator(s):

Strengths

- A senior biostatistician has been added as a co-mentor.

Weaknesses

- *Reviewer did not provide any comments regarding weaknesses*

5. Environment:

Strengths

- Outstanding

Weaknesses

- *Reviewer did not provide any comments regarding weaknesses*

Training in the Responsible Conduct of Research: Acceptable

Comments on Format (Required):

- Acceptable

Comments on Subject Matter (Required):

- Acceptable

Comments on Faculty Participation (Required):

- Acceptable

Comments on Duration (Required):

- Acceptable

Comments on Frequency (Required):

- Acceptable

Authentication of Key Biological and/or Chemical Resources: Not applicable (No Relevant Resources)

Protections for Human Subjects: Acceptable Risks and Adequate Protections

- Concern with asking control subjects not to exercise in Aim 2.

Data and Safety Monitoring Plan (Applicable for Clinical Trials Only): Acceptable

Inclusion of Women, Minorities, and Ages Across the Lifespan:

- Sex/Gender: G1A: Both sexes used: Scientifically Acceptable
- Race/Ethnicity: M1A: Minority and non-minority: Scientifically Acceptable
- For NIH-Defined Phase III trials, Plans for valid design and analysis:
- Inclusion/Exclusion Across the Lifespan: C3A: No Children Included (Age ≥ 18); Scientifically Acceptable

Vertebrate Animals: Not applicable (No Vertebrate Animals)

Biohazards: Not applicable (No Biohazards)

Resubmission: Acceptable

Select Agents: Not applicable (No Select Agents)

Resource Sharing Plans: Not applicable (No Relevant Resources)

Budget and Period of Support: Recommend as Requested

THE FOLLOWING SECTIONS WERE PREPARED BY THE SCIENTIFIC REVIEW OFFICER TO SUMMARIZE THE OUTCOME OF DISCUSSIONS OF THE REVIEW COMMITTEE, OR REVIEWERS' WRITTEN CRITIQUES, ON THE FOLLOWING ISSUES:

PROTECTION OF HUMAN SUBJECTS (Resume): ACCEPTABLE

INCLUSION OF WOMEN PLAN (Resume): ACCEPTABLE. G1A

INCLUSION OF MINORITIES PLAN (Resume): ACCEPTABLE. M1A

INCLUSION OF ACROSS THE LIFESPAN (Resume): ACCEPTABLE. C3A

VERTEBRATE ANIMAL (Resume): NOT APPLICABLE

BIOHAZARD COMMENT: NOT APPLICABLE

MODEL ORGANISM SHARING PLAN: NOT APPLICABLE

DATA/RESOURCE SHARING: ACCEPTABLE

TRAINING IN THE RESPONSIBLE CONDUCT OF RESEARCH: ACCEPTABLE

AUTHENTICATION OF KEY BIOLOGICAL AND/OR CHEMICAL RESOURCES: NOT APPLICABLE

COMMITTEE BUDGET RECOMMENDATIONS: RECOMMENDED AS REQUESTED.

Footnotes for 1 K23 NS123506-01A1; PI Name: Luthra, Nijee

NIH has modified its policy regarding the receipt of resubmissions (amended applications).See Guide Notice NOT-OD-18-197 at https://grants.nih.gov/grants/guide/notice-files/NOT-OD-18-197.html. The impact/priority score is calculated after discussion of an application by averaging the overall scores (1-9) given by all voting reviewers on the committee and multiplying by 10. The criterion scores are submitted prior to the meeting by the individual reviewers assigned to an application, and are not discussed specifically at the review meeting or calculated into the overall impact score. Some applications also receive a percentile ranking. For details on the review process, see http://grants.nih.gov/grants/peer_review_process.htm#scoring.

MEETING ROSTER

NST-1 Study Section
Neurological Sciences Training Initial Review Group
NATIONAL INSTITUTE OF NEUROLOGICAL DISORDERS AND STROKE
NST-1

NST-1
01/31/2022 - 02/01/2022

Notice of NIH Policy to All Applicants: Meeting rosters are provided for information purposes only. Applicant investigators and institutional officials must not communicate directly with study section members about an application before or after the review. Failure to observe this policy will create a serious breach of integrity in the peer review process, and may lead to actions outlined in NOT-OD-14-073 at https://grants.nih.gov/grants/guide/notice-files/NOT-OD-14-073.html, NOT-OD-15-106 at https://grants.nih.gov/grants/guide/notice-files/NOT-OD-15-106.html, and NOT-OD-18-115 at https://grants.nih.gov/grants/guide/notice-files/NOT-OD-18-115.html, including removal of the application from immediate review.

CHAIRPERSON(S)

DOUGHERTY, PATRICK M, PHD
PROFESSOR
DEPARTMENT OF PAIN MEDICINE
THE UNIVERSITY OF TEXAS
HOUSTON, TX 77030

FURIE, KAREN L, MD
PROFESSOR & CHAIR
DEPARTMENT OF NEUROLOGY
RHODE ISLAND HOSPITAL
PROVIDENCE, RI 02903

MEMBERS

AMIN-HANJANI, SEPIDEH, MD
PROFESSOR
DEPARTMENT OF NEUROSURGERY
UNIVERSITY OF ILLINOIS, CHICAGO
CHICAGO, IL 60612

AOUIZERAT, BRADLEY E, PHD
PROFESSOR
DEPARTMENT OF ORAL AND MAXILLOFACIAL SURGERY
NEW YORK UNIVERSITY
NEW YORK, NY 10010

BECHER, OREN JOSH, MD
PROFESSOR
DEPARTMENT OF PEDIATRICS AND ONCOLOGICAL SCIENCES
ICAHN SCHOOL OF MEDICINE MOUNT SINAI
NEW YORK, NY 60611

BIEBER, ALLAN JAMES, PHD
ASSOCIATE PROFESSOR
DEPARTMENT OF NEUROLOGY
DARTMOUTH COLLEGE
HANOVER, NH 03755

CARMEL, JASON BRANT, MD, PHD
ASSOCIATE PROFESSOR
DEPARTMENTS OF NEUROLOGY AND OTHOPEDICS
COLUMBIA UNIVERSITY MEDICAL CENTER
NEW YORK, NY 10032

CASSARLY, CHRISTY, PHD
ASSISTANT PROFESSOR
DEPARTMENT OF PUBLIC HEALTH SCIENCES
MEDICAL UNIVERSITY OF SOUTH CAROLINA
CHARLESTON, SC 29425

CHALAK, LINA F, MD
PROFESSOR
DEPARTMENT OF PEDIATRICS, PSYCHIATRY, AND NEONATAL-PERINATAL MEDICINE
UNIVERSITY OF TEXAS SOUTHWESTERN MEDICAL CENTER
DALLAS, TX 75390

CHANG, ANNE-MARIE, PHD
ASSOCIATE PROFESSOR
DEPARTMENT OF BIOBEHAVIORAL HEALTH
PENNSYLVANIA STATE UNIVERSITY
STATE COLLEGE, PA 16802

DEY, MAHUA, MD
ASSISTANT PROFESSOR
DEPARTMENT OF NEUROLOGICAL SURGERY
UNIVERSITY OF WISCONSIN
MADISON, WI 53792

EMERSON, CHARLES P. JR, PHD
PROFESSOR
DEPARTMENT OF NEUROLOGY
UNIVERSITY OF MASSACHUSETTS MEDICAL SCHOOL
WORCESTER, MA 01655

ESS, KEVIN C, MD, PHD
ASSOCIATE PROFESSOR OF PEDIATRICS
DIVISION OF PEDIATRIC NEUROLOGY
VANDERBILT UNIVERSITY MEDICAL CENTER
NASHVILLE, TN 37232

GENDELMAN, HOWARD E, MD
PROFESSOR AND CHAIR
DEPARTMENT OF PHARMACOLOGY AND EXPERIMENTAL NEUROSCIENCE
UNIVERSITY OF NEBRASKA MEDICAL CENTER
OMAHA, NE 68198

GERHARDT, GREG A., PHD
PROFESSOR & SCIENTIFIC DIRECTOR
DEPT. OF NEUROSCIENCE, NEUROLOGY & NEUROSURGERY
UNIVERSITY OF KENTUCKY MEDICAL CENTER
LEXINGTON, KY 40536-0298

GHILARDI, M. FELICE MARINA, MD
PROFESSOR
DEPARTMENT OF PHYSIOLOGY, PHARMACOLOGY, NEUROSCIENCE AND NEUROLOGY
CUNY MEDICAL SCHOOL
NEW YORK, NY 10031

GOYAL, MANU S, MD
ASSOCIATE PROFESSOR
DEPARTMENT OF RADIOLOGY
WASHINGTON UNIVERSITY
ST. LOUIS, MO 63144

GREGORY, SIMON G, PHD
PROFESSOR
DEPARTMENTS OF MEDICINE, NEUROLOGY, & MOLECULAR GENETICS AND MICROBIOLOGY
DUKE UNIVERSITY MEDICAL CENTER
DURHAM, NC 27710

GUERRERO-CAZARES, HUGO, MD, PHD
ASSOCIATE PROFESSOR
DEPARTMENT OF NEUROSURGERY
MAYO CLINIC
JACKSONVILLE, FL 32082

GUTMANN, LAURIE, MD
PROFESSOR & CHAIR
DEPARTMENT OF NEUROLOGY
INDIANA UNIVERSITY SCHOOL OF MEDICINE
INDIANAPOLIS, IN 46202

HEATHCOCK, JILL C., PHD
ASSOCIATE PROFESSOR
SCHOOL OF HEALTH AND REHABILITATION SCIENCE
THE OHIO STATE UNIVERSITY
COLUMBUS, OH 43210

JINNAH, HYDER A, MD, PHD
PROFESSOR
DEPARTMENTS OF NEUROLOGY AND HUMAN GENETICS
EMORY UNIVERSITY
ATLANTA, GA 30322

KORNBLUM, HARLEY IAN, PHD, MD
PROFESSOR
DEPTS OF PSYCHIATRY, PHARMACOLOGY, AND PEDIATRICS
UCLA SEMEL INST. FOR NEUROSCIENCE & HUMAN BEHAVIOR
LOS ANGELES, CA 90095

LATHIA, JUSTIN D, PHD
PROFESSOR AND VICE CHAIR
DEPARTMENTS OF MOLECULAR MEDICINE AND CARDIOVASCULAR AND METABOLIC SCIENCES
CLEVELAND CLINIC LERNER COLLEGE OF MEDICINE
CLEVELAND, OH 44195

LHATOO, SAMDEN, MD
PROFESSOR
DEPARTMENT OF NEUROLOGY
UNIVERSITY OF TEXAS AT HOUSTON
HOUSTON, TX 77030

LITVAN, IRENE, MD
DISTINGUISHED PROFESSOR
DEPARTMENT OF NEUROLOGY
UNIVERSITY OF CALIFORNIA, SAN DIEGO
LA JOLLA, CA 92093

MACK, WILLIAM J, MD
PROFESSOR OF NEUROLOGICAL SURGERY
DEPARTMENT OF NEUROSURGERY
UNIVERSITY OF SOUTHERN CALIFORNIA
LOS ANGELES, CA 90033

MACMILLAN, CAROL, MD
ASSOCIATE PROFESSOR
DEPARTMENT OF PEDIATRICS
UNIVERSITY OF CHICAGO
CHICAGO, IL 60637

MARCO, ELYSA JILL, MD
PROFESSOR AND MEDICAL DIRECTOR
CLINICAL EXECUTIVE FOR NEURODEVELOPMENTAL MEDICINE
CORTICA INC.
SAN RAFAEL, CA 94143

MARSH, ERIC D, MD, PHD
ASSOCIATE PROFESSOR
DIVISION OF CHILD NEUROLOGY
CHILDREN'S HOSPITAL OF PHILADELPHIA
PHILADELPHIA, PA 19104

MCFARLAND, NIKOLAUS RENZ, MD, PHD
ASSOCIATE PROFESSOR
DEPARTMENT OF NEUROLOGY
UNIVERSITY OF FLORIDA
GAINESVILLE, FL 32610

MIAO, HONGYU, PHD
ASSOCIATE PROFESSOR
DEPARTMENT OF BIOSTATISTICS
UNIVERSITY OF TEXAS HEALTH SCIENCE CENTER, HOUSTON
HOUSTON, TX 77030

MINK, JONATHAN W., MD, PHD
PROFESSOR
DEPARTMENT OF NEUROLOGY
UNIVERSITY OF ROCHESTER MEDICAL CENTER
ROCHESTER, NY 14642

O'BRYANT, SID E, PHD
PROFESSOR & ENDOWED CHAIR
DEPARTMENT OF PHARMACOLOGY & NEUROSCIENCE
UNIVERSITY OF NORTH TEXAS DALLAS FORT WORTH
FORT WORTH, TX 76107

OJEMANN, JEFFREY G, MD
VICE CHAIRMAN AND PROFESSOR
DEPARTMENT OF NEUROLOGICAL SURGERY
UNIVERSITY OF WASHINGTON
SEATTLE, WA 98195

PANDEY, UDAI B, PHD
ASSOCIATE PROFESSOR
DEPARTMENT OF PEDIATRICS
CHILDREN'S HOSPITAL OF PITTSBURGH
PITTSBURGH, PA 15224

PAUL, ROBERT H, PHD
PROFESSOR AND DIRECTOR
DEPARTMENT OF PSYCHOLOGICAL SCIENCES
UNIVERSITY OF MISSOURI, ST LOUIS
ST LOUIS, MO 02906

PETERS, JURRIAAN M., MD
ASSISTANT PROFESSOR
DEPARTMENT OF NEUROLOGY
HARVARD MEDICAL SCHOOL
BOSTON, MA 02115

PLEASURE, SAMUEL JEREMY, PHD, MD
PROFESSOR AND GLENN W. JOHNSON, JR. MEMORIAL ENDOWED CHAIR
DEPARTMENT OF NEUROLOGY
UNIVERSITY OF CALIFORNIA, SAN FRANCISCO
SAN FRANCISCO, CA 94158

PODURI, ANNAPURNA, MD
PROFESSOR
DEPARTMENT OF NEUROLOGY
HARVARD MEDICAL SCHOOL
BOSTON, MA 02115

RHO, JONG MIN, MD
PROFESSOR
DEPARTMENT OF NEUROSCIENCES, PEDIATRICS & PHARMACOLOGY
UNIVERSITY OF CALIFORNIA SAN DIEGO
LA JOLLA, CA 92093

ROBERTSON, COURTNEY L, MD
ASSOCIATE PROFESSOR
DEPARTMENT OF ANESTHESIA & CRITICAL CARE MEDICINE
JOHN HOPKINS SCHOOL OF MEDICINE
BALTIMORE, MD 21287-4904

ROBINSON, CATRINA SIMS, PHD
ASSOCIATE PROFESSOR
DEPARTMENT OF NEUROLOGY
MEDICAL UNIVERSITY OF SOUTH CAROLINA
CHARLESTON, SC 29461

ROBINSON-PAPP, JESSICA, MD
ASSOCIATE PROFESSOR
DEPARTMENT OF NEUROLOGY
ICAHN SCHOOL OF MEDICINE AT MOUNT SINAI
NEW YORK, NY 10029

SANSING, LAUREN H, MD
ASSOCIATE PROFESSOR
DEPARTMENT OF NEUROLOGY
YALE UNIVERSITY
NEW HAVEN, CT 06511

SARATSIS, AMANDA MUHS, MD
ASSOCIATE PROFESSOR
DEPARTMENT OF NEUROSURGERY
LURIE CHILDREN'S HOSPITAL
NORTHWESTERN UNIVERSITY
CHICAGO, IL 60611

SHOYKHET, MICHAEL, MD, PHD
ASSOCIATE PROFESSOR
DEPARTMENT OF CRITICAL CARE MEDICINE
CHILDREN'S RESEARCH INSTITUTE
WASHINGTON, DC 20010

SIEGENTHALER, JULIE, PHD
ASSOCIATE PROFESSOR
DEPARTMENT OF PEDIATRICS
UNIVERSITY OF COLORADO
AURORA, CO 80045

STALEY, KEVIN J., MD
PROFESSOR
DEPARTMENT OF NEUROLOGY
MASSACHUSETTS GENERAL HOSPITAL
BOSTON, MA 02114

TIMMONS, SHELLY D, MD, PHD
PROFESSOR & CHAIR
DEPARTMENT OF NEUROLOGICAL SURGERY
INDIANA UNIVERSITY SCHOOL OF MEDICINE
INDIANAPOLIS, IN 46202

TRANEL, DANIEL T., PHD
PROFESSOR OF NEUROLOGY
DEPARTMENT OF PSYCHOLOGICAL AND BRAIN SCIENCES
UNIVERSITY OF IOWA
IOWA CITY, IA 52242

WILLIS, ALLISON, MD
ASSOCIATE PROFESSOR
DEPARTMENTS OF NEUROLOGY AND EPIDEMIOLOGY
UNIVERSITY OF PENNSYLVANIA
PHILADELPHIA, PA 19104

ZHANG, JOHN H, MD, PHD
PROFESSOR & VICE CHAIR
DEPARTMENT OF BASIC SCIENCES
LOMA LINDA UNIVERSITY
LOMA LINDA, CA 92350

SCIENTIFIC REVIEW OFFICER

BENZING, WILLIAM C., PHD
SCIENTIFIC REVIEW OFFICER
SCIENTIFIC REVIEW BRANCH
DIVISION OF EXTRAMURAL RESEARCH
NATIONAL INSTITUTE OF NEUROLOGICAL DISORDERS
AND STROKE, NIH
BETHESDA, MD 20892

EXTRAMURAL SUPPORT ASSISTANT

WATTS, DEVONA LAQUELL
EXTRAMURAL SUPPORT ASSISTANT
NINDS
SCIENTIFIC REVIEW BRANCH
6001 EXECUTIVE BLVD, SUITE 3208
ROCKVILLE, MD 20852

Consultants are required to absent themselves from the room during the review of any application if their presence would constitute or appear to constitute a conflict of interest.
